# Honey microbiota, methods for determining the microbiological composition and the antimicrobial effect of honey – A review

**DOI:** 10.1016/j.fochx.2024.101524

**Published:** 2024-06-04

**Authors:** Liliana Luca, Daniela Pauliuc, Mircea Oroian

**Affiliations:** a Suceava-Botoșani Regional Innovative Bioeconomy Cluster Association, 720229 Suceava, Romania; b Faculty of Food Engineering, Stefan cel Mare University of Suceava, 720229 Suceava, Romania

**Keywords:** Honey, Microbiota, Pathogenic microorganisms, Antimicrobial effect

## Abstract

Honey is a natural product used since ancient times due to its taste, aroma, and therapeutic properties (antibacterial, antiviral, anti-inflammatory, and antioxidant activity). The purpose of this review is to present the species of microorganisms that can survive in honey and the effect they can have on bees and consumers. The techniques for identifying the microorganisms present in honey are also described in this study. Honey contains bacteria, yeasts, molds, and viruses, and some of them may present beneficial properties for humans. The antimicrobial effect of honey is due to its acidity and high viscosity, high sugar concentration, low water content, the presence of hydrogen peroxide and non-peroxidase components, particularly methylglyoxal (MGO), phenolic acids, flavonoids, proteins, peptides, and non-peroxidase glycopeptides. Honey has antibacterial action (it has effectiveness against bacteria, e.g. *Escherichia coli*, *Pseudomonas aeruginosa*, *Staphylococcus aureus*, and *Acinetobacter,* etc.), antifungal (effectiveness against *Candida* spp., *Aspergillus* spp.*, Fusarium* spp.*, Rhizopus* spp.*,* and *Penicillium* spp.), antiviral (effectiveness against SARS-CoV-2, Herpes simplex virus type 1, Influenza virus A and B, Varicella zoster virus), and antiparasitic action (effectiveness against *Plasmodium berghei, Giardia* and *Trichomonas, Toxoplasma gondii*) demonstrated by numerous studies that are comprised and discussed in this review.

## Highlights

• Honey can have beneficial or pathogenic microorganisms for bees and humans.
• The antimicrobial action is determined by the H_2_O_2_ and non-peroxidase components.
• Honey has antibacterial action, antifungal, antiviral and, antiparasitic.

## 1 Introduction

Honey is the naturally sweet substance produced by *Apis mellifera* bees that gather plant nectar, secretions from living plant parts, or excretions from insects that feed by sucking on plants. The bees gather, transform, and combine these elements with their unique substances, then proceed to dehydrate, and store them in honeycombs for the maturation process (Council, 2002). Honey is well known for its health benefits, having antibacterial, antioxidant, anti-inflammatory, antimutagenic, anticarcinogenic, and, bacteriostatic qualities (Albaridi, 2019; Alvarez-Suarez et al., 2014), and it also helps to cure wounds and sunburns (Ahmed et al., 2018; Jia et al., 2020; Samarghandian et al., 2017). In its composition, honey has minor compounds, like enzymes, minerals, amino acids, polyphenols, vitamins, and major compounds, such as monosaccharides (glucose and fructose) (da Silva et al., 2016). The natural microbiological and physicochemical properties of honey vary depending on its botanical origin (plant species visited by bees), seasonal differences, climatic conditions, geographical origin and applied processes (Seraglio et al., 2019; Mărgăoan et al., 2021; Wright et al., 2018; Martinello & Mutinelli, 2021; Alqarni et al., 2016; Matzen et al., 2018a, Matzen et al., 2018b; Machado De-Melo et al., 2018; Jia et al., 2020).

Honey can transfer many of the therapeutic qualities of plants, so it could be used as a way of delivering these qualities (Brudzynski, 2021). Therefore, honey is regarded as a vital natural resource that can be utilized in new treatments without causing the side effects that frequently go along with the use of synthetic chemical drugs (Ahmed et al., 2018; Szweda, 2017). Because honey can contain a variety of antimicrobial and prebiotic compounds, depending on the source of the flowers, it is regarded as a functional food that is good for both human and animal health (Grabowski & Klein, 2017), especially in favor of gut microbiota balanced composition (Mohan et al., 2017). However, for the honey to have the expected effects, a strict compliance to certification and quality assurance procedures is necessary (Iurlina & Fritz, 2005).

Honey contains organic acids, which lower its pH value, water activity (about 17.3%), hydrogen peroxide (from the action of glucose oxidase), and has a high osmotic pressure (from low water activity) that influences the growth of microorganisms. Also, the presence of chemical agents – e.g. phenolic acids, benzyl alcohol, lysozyme, volatile substances, high carbon/nitrogen ratio, and low protein content inhibits the multiplication of microorganisms in honey (Grabowski & Klein, 2017; Silva et al., 2017). Currently, many researchers analyzed the physical and chemical properties of honey and its ability to act against various microorganisms responsible for numerous human ailments was demonstrated (Nolan et al., 2019; Szweda, 2017). Microorganisms in honey have the potential to affect its safety and quality (Al-Waili et al., 2012). The need to analyze honey microbiota will continue to increase as new ways to use honey and new technologies are developed. Environmental factors determine how hygienic honey is and these factors can be categorized into the areas around the hive that bees visit (dust, air, water, flowers, etc.), the hive itself, the bee body cavities where honey is produced, as well as the circumstances surrounding honey harvest and packaging (Vázquez-Quiñones et al., 2018).

The purpose of this review is to present a comprehensive evaluation of the scientific data that characterize the microbiological properties of honey and support its use as a viable antimicrobial substitute against drug-resistant bacteria, either on its own or in conjunction with currently available chemotherapeutic agents. This paper briefly describes the microbiology of honey and methods of its analysis for the purpose of sanitary quality and food safety certification, alongside primary research that proves the efficacy of honey against bacteria with resistance.

## 2 The microbiota present in honey

The microbiological composition of honey is very diverse and is summarized in Table 1, Table 2, Table 3. The microorganisms found in honey originate from various sources and some authors classify these sources into primary and secondary sources (Santorelli et al., 2023). The primary sources are represented by the intrinsic microorganisms found in the gut of the bees, by the microorganisms collected from the environment that the bees explore (soil, air, dust, plants, and flowers) to gather the ingredients needed to produce honey (nectar, pollen, and plant sap), and by the microorganisms inside the hive, etc. Secondary sources are microorganisms that can contaminate honey during honey extraction, handling (processing facilities, equipment, and personnel), and honey storage (Silva et al., 2017; Vázquez-Quiñones et al., 2018).

**Table 1:** Bacteria that can be present in honey (clade - *Terrabacteria* group).

Phylum	Class	Order	Family	Genus	Reference
*Proteobacteria /* *Pseudomonadota*	*Alphaproteobacteria*	*Sphingomonadales*	*Zymomonadaceae*	*Zymomonas*	Khan et al. (2020); Silva et al. (2017); Bovo et al. (2018)
		*Rhodospirillales*	*Acetobacteraceae*	*Parasaccharibacter*	Silva et al. (2017); Bovo et al. (2018)
				*Asia*	Xiong et al. (2023); Stavropoulou et al. (2023)
				*Saccharibacter*	Balzan et al. (2020)
				*Bombella*	Xiong et al. (2023)
				*Gluconobacter*	Khan et al. (2020); Silva et al. (2017); Brudzynski (2021); Begum et al. (2015)
		*Hyphomicrobiales*	*Nitrobacteraceae*	*Bradyrhizobium*	Stavropoulou et al. (2023)
	*Gammaproteobacteria*	*Pseudomonadales*	*Pseudomonadaceae*	*Pseudomonas*	Vázquez-Quiñones et al. (2018); Xiong et al. (2023); Stavropoulou et al. (2023); Brudzynski (2021)
	*Gammaproteobacteria*	*Enterobacteriales*	*Enterobacteriacea*	*Enterobacter*	Xiong et al. (2023); Vázquez-Quiñones et al. (2018); Ngalimat et al., 2019a, Ngalimat et al., 2019b; Balzan et al. (2020)
				*Escherichia*	Silva et al. (2017); Ngalimat et al., 2019a, Ngalimat et al., 2019b; Adadi and Obeng, 2017a, Adadi and Obeng, 2017b
				*Citrobacter*	Vázquez-Quiñones et al. (2018); Khan et al. (2017); Xiong et al. (2023)
				*Cedecea*	Xiong et al. (2023)
				*Vagococcus*	Xiong et al. (2023)
				*Raoultella*	Xiong et al. (2023)
				*Salmonella*	Bovo et al. (2018); Fernández et al. (2017)
				*Shigella*	Balzan et al. (2020); Fernández et al. (2017); Adadi and Obeng, 2017a, Adadi and Obeng, 2017b
				*Klebsiella*	Silva et al. (2017); Brudzynski (2021); Sinacori et al. (2014)
	*Gammaproteobacteria*		*Erwiniaceae*	*Erwinia*	Vázquez-Quiñones et al. (2018); Xiong et al. (2023); Bovo, Utzeri, Ribani, Cabbri and Fontanesi, 2020a, Bovo, Utzeri, Ribani, Cabbri and Fontanesi, 2020b
				*Pantoea*	Ngalimat et al., 2019a, Ngalimat et al., 2019b; Beux et al., 2022a, Beux et al., 2022b; Bovo et al. (2018)
			*Pectobacteriaceae*	*Lonsdalea*	Stavropoulou et al. (2023)
			*Yersiniaceae*	*Serratia*	Xiong et al. (2023)
			*Morganellaceae*	*Providencia*	Xiong et al. (2023); Khan et al. (2017)
				*Morganella*	Xiong et al. (2023); Khan et al. (2017)
				*Proteus*	Xiong et al. (2023); Khan et al. (2017)
		*Moraxellales*	*Moraxellaceae*	*Acinetobacter*	Vázquez-Quiñones et al. (2018); Balzan et al. (2020)
		*Oceanospirillales*	*Halomonadaceae*	*Zymobacter*	Stavropoulou et al. (2023)
				*Halomonadas*	Balzan et al. (2020)
		*Orbales*	*Orbaceae*	*Gilliamella*	Silva et al. (2017); Bovo et al. (2018)
				*Frischella*	Silva et al. (2017); Bovo et al. (2018)
		*Vibrionales*	*Vibrionaceae*	*Vibrio*	Balzan et al. (2020)
		*Xanthomonadales*	*Xanthomonadaceae*	*Lysobacter*	Stavropoulou et al. (2023)
				*Stenotrophomonas*	Xiong et al. (2023)
	*Betaproteobacteria*	*Neisseriales*	*Neisseriaceae*	*Snodgrassella*	Silva et al. (2017)
		*Burkholderiale*	*Alcaligenaceae*	*Achromobacter*	Al-Waili et al. (2012)
				*Alcaligenes*	Al-Waili et al. (2012)
			*Comamonadaceae*	*Delftia*	Xiong et al. (2023)
*Campylobacterota*	*Epsilonproteobacteria*	*Campylobacterales*	*Campylobacteraceae*	*Campylobacter*	Balzan et al. (2020)
*Bacteroidota*	*Flavobacteriia*	*Flavobacteriales*	*Flavobacteriaceae*	*Flavobacterium*	Khan et al. (2017); Vázquez-Quiñones et al. (2018)
	*Bacteroidia*	*Bacteroidales*	*Bacteroidaceae*	*Bacteroides*	Silva et al. (2017)
*Mycoplasmatota*	*Mollicutes*	*Entomoplasmatales*	*Spiroplasmataceae*	*Spiroplasma*	Bovo et al. (2018)
*Actinobacteria/ Actinomycetota*	*Actinomycetes*	*Actinomycetales*	*Actinomycetaceae*	*Actinomyces*	Silva et al. (2017)
			*Micrococcaceae*	*Micrococcus*	Al-Waili et al. (2012); Ngalimat et al., 2019a, Ngalimat et al., 2019b; Pomastowski et al., 2019a, Pomastowski et al., 2019b
	*Actinomycetes*	*Bifidobacteriales*	*Bifidobacteriaceae*	*Bifidobacterium*	Brudzynski (2021); Nowak et al. (2021); Ebrahimi et al. (2021); Jia et al. (2020); Abdelbary et al. (2017)
		*Micrococcales*	*Micrococcaceae*	*Micrococcus*	Adadi and Obeng, 2017a, Adadi and Obeng, 2017b; Mustar and Ibrahim (2022); Pomastowski et al., 2019a, Pomastowski et al., 2019b
			*Microbacteriaceae*	*Microbacterium*	Pajor et al. (2018); Khan et al. (2017); Bovo et al. (2018); Brudzynski (2021)
			*Brevibacteriaceae*	*Brevibacterium*	Al-Waili et al. (2012)
		*Propionibacteriales*	*Propionibacteriaceae*	*Propionibacterium*	Balzan et al. (2020)
		*Kitasatosporale*	*Streptomycetaceae*	*Streptomyces*	Ngalimat et al., 2019a, Ngalimat et al., 2019b
		*Mycobacteriales*	*Corynebacteriaceae*	*Corynebacterium*	Vázquez-Quiñones et al. (2018); Balzan et al. (2020)
*Bacillota/ Firmicutes*	*Bacilli*	*Bacillales*	*Bacillaceae*	*Bacillus*	Beux et al., 2022a, Beux et al., 2022b; Silva et al. (2017); Xiong et al. (2023)
				*Lysinibacillus*	Beux et al., 2022a, Beux et al., 2022b; Brudzynski (2021); Pomastowski et al., 2019a, Pomastowski et al., 2019b; Pajor et al. (2018)
				*Oceanobacillus*	Brudzynski (2021)
			*Paenibacillaceae*	*Paenibacillus*	Balzan et al. (2020); Beux et al., 2022a, Beux et al., 2022b; Brudzynski (2021); Kędzierska-Matysek et al. (2023)
				*Brevibacillus*	Brudzynski (2021); Pomastowski et al., 2019a, Pomastowski et al., 2019b
			*Staphylococcaceae*	*Staphylococcus*	Silva et al. (2017); Ngalimat et al., 2019a, Ngalimat et al., 2019b; Brudzynski (2021); Pajor et al. (2018); Pomastowski et al., 2019a, Pomastowski et al., 2019b
			*Listeriaceae*	*Listeria*	Silva et al. (2017); Brudzynski (2021)
			*Planococcaceae*	*Planomicrobium*	Khan et al. (2017)
			*Bacillales Family XII. Incertae Sedis;*	*Exiguobacterium*	Khan et al. (2017)
	*Bacilli*	*Lactobacillales*	*Lactobacillaceae*	*Lactobacillus*	Khan et al. (2020); Abadi et al. (2023); Mustar and Ibrahim (2022); Lashani et al. (2020); Razmgah et al. (2016); Xiong et al. (2023);Vázquez-Quiñones et al. (2018); Feizabadi et al. (2021)
				*Pediococcus*	Vázquez-Quiñones et al. (2018); Balzan et al. (2020); Yan et al. (2019)
				*Fructobacillus*	Balzan et al. (2020); Syed Yaacob et al. (2018); Takatani and Endo (2021)
				*Leuconostoc*	Beux et al., 2022a, Beux et al., 2022b; Vázquez-Quiñones et al. (2018); Mustar and Ibrahim (2022)
				*Oenococcus*	Brudzynski (2021)
				*Weissella*	Brudzynski (2021)
			*Streptococcaceae*	*Lactococcus*	Vázquez-Quiñones et al. (2018); Seraglio et al. (2019); Sinacori et al. (2014); Mustar and Ibrahim (2022)
				*Streptococcus*	Silva et al. (2017); Balzan et al. (2020); Brudzynski (2021); Stavropoulou et al. (2023)
			*Enterococcaceae*	*Enterococcus*	Silva et al. (2017); Balzan et al. (2020); Santorelli et al. (2023); Feizabadi et al. (2021); Sinacori et al. (2014)
				*Melissococcus*	Brudzynski (2021); Bovo et al. (2018)
				*Aerococcus*	Brudzynski (2021); Sinacori et al. (2014)
				*Carnobacterium*	Brudzynski (2021)
			*Pediococcaceae*	*Pediococcus*	Brudzynski (2021); Bulgasem et al. (2016)
	*Clostridia*	*Eubacteriales/* *Clostridiales*	*Clostridiaceae*	*Clostridium*	Al-Waili et al. (2012); Vázquez-Quiñones et al. (2018); Xiong et al. (2023); Balzan et al. (2020); Maikanov et al. (2019); Grenda et al. (2018); Wojtacka et al. (2016); Grigoryan (2016); Guran et al. (2023)
			*Oscillospiraceae*	*Faecalibacterium*	Balzan et al. (2020)
			*Lachnospiraceae*	*Coprococcus*	Balzan et al. (2020)
				*Tyzzerella*	Xiong et al. (2023)
*Deinococcota*	*Deinococci*	*Thermales*	*Thermaceae*	*Meiothermus*	Stavropoulou et al. (2023)
*Mycoplasmatota*	*Mollicutes*	*Entomoplasmatales*	*Spiroplasmataceae*	*Spiroplasma*	Schwarz et al. (2014)

**Table 2:** Fungi that can be present in honey (*Eumycota* kingdom).

Phylum	Class	Order	Family	Genus	References
*Ascomycota*	*Ascomycota incertae sedis* *Eurotiomycetes*			*Oosporidium*	Silva et al. (2017)
		*Onygenales*	*Ascosphaeraceae*	*Ascosphaera*	Al-Waili et al. (2012); Grabowski and Klein (2017); Rodríguez-Andrade et al. (2019); Xiong et al. (2023); Ziuzia et al. (2023)
			*Eremascaceae*	*Eremascus*	Rodríguez-Andrade et al. (2019)
			*Onygenales incertae sedis*	*Helicoarthrosporum*	Rodríguez-Andrade et al. (2019)
				*Trichosporum*	Roxo et al. (2023)
				*Strongyloarthrosporum*	Rodríguez-Andrade et al. (2019)
		*Eurotiales*	*Aspergillaceae*	*Aspergillus*	Xiong et al. (2023); Silva et al. (2017); Rodríguez-Andrade et al. (2019); Brudzynski (2021); Kostić et al. (2017); Cinar and Cinar (2021); Rašić et al. (2019); Roxo et al. (2023)
				*Penicillium*	Silva et al., 2017); Brudzynski (2021); Sinacori et al. (2014); Rodríguez-Andrade et al. (2019); Bovo, Utzeri, Ribani, Cabbri and Fontanesi, 2020a, Bovo, Utzeri, Ribani, Cabbri and Fontanesi, 2020b; Sinkevičienė and Amšiejus (2019)
				*Monascus*	Brudzynski (2021); Rodríguez-Andrade et al. (2019)
				*Eurotium*	Cinar and Cinar (2021)
				*Emericella*	Silva et al. (2017)
			*Trichocomaceae*	*Talaromyces*	Brudzynski (2021); Rodríguez-Andrade et al. (2019)
			*Thermoascaceae*	*Paecilomyces*	Kačániová et al. (2012)Rodríguez-Andrade et al. (2019); Cinar and Cinar (2021)
	*Saccharomycetes*	*Saccharomycetales*	*Lipomycetaceae*	*Lipomyces*	Al-Waili et al. (2012)
			*Pichiaceae*	*Pichia*	Silva et al. (2017); Echeverrigaray et al., 2021, Wei et al., 2022
				*Hansenula*	Silva et al. (2017); Ziuzia et al. (2023); Grabowski and Klein (2017)
	*Saccharomycetes*	*Saccharomycetales*		*Brettanomyces/Dekkera*	Echeverrigaray et al. (2021)
				*Starmerella*	Ziuzia et al. (2023); Xiong et al. (2023); Buzzini et al. (2018); Sinacori et al. (2014); Silva et al. (2017)
			*Debaryomycetaceae*	*Candida*	Beux et al., 2022a, Beux et al., 2022b; Balzan et al. (2020); Ziuzia et al. (2023); Echeverrigaray et al., 2021, Wei et al., 2022; Rodríguez-Andrade et al. (2019); Brudzynski (2021)
				*Torulopsis*	Silva et al. (2017); Rodríguez-Andrade et al. (2019); Kędzierska-Matysek et al. (2023); Brudzynski (2021)
				*Wickerhamia/Kloeckera*	Echeverrigaray et al. (2021)
				*Debaryomyces*	Silva et al. (2017), Echeverrigaray et al., 2021, Sinacori et al., 2014, Ziuzia, Janiec, Wróbel-Kwiatkowska, Lazar, & Rakicka-Pustułka, 2023
			*Saccharomycetaceae*	*Zygosaccharomyces*	Beux et al., 2022a, Beux et al., 2022b; Silva et al. (2017); Balzan et al. (2020); Ziuzia et al. (2023); Echeverrigaray et al. (2021); Xiong et al. (2023); Buzzini et al. (2018); Grabowski and Klein (2017)
				*Torulaspora*	Echeverrigaray et al. (2021)
				*Eremothecium*	Wei et al. (2022)
				*Saccharomyces*	Brudzynski (2021); Xiong et al. (2023); Vázquez-Quiñones et al. (2018); Rodríguez-Andrade et al. (2019)
			*Saccharomycodaceae*	*Hanseniaspora*	Balzan et al. (2020); Grabowski and Klein (2015); Buzzini et al. (2018)
			*Metschnikowiaceae*	*Clavispora*	Xiong et al. (2023); Wei et al. (2022)
			*Dipodascaceae*	*Yarrowia*	Ziuzia et al. (2023); Xiong et al. (2023); Grabowski and Klein (2017); Kędzierska-Matysek et al. (2023)
			*Trichomonascaceae*	*Wickerhamiella*	Echeverrigaray et al. (2021)
				*Blastobotrys*	Rodríguez-Andrade et al. (2019)
			*Phaffomycetaceae*	*Wickerhamomyces*	Echeverrigaray et al. (2021); Xiong et al. (2023); Wei et al. (2022)
				*Barnettozyma*	Wei et al. (2022)
				*Cyberlindnera*	Brudzynski (2021)
		*Xylariales*	*Hypoxylaceae*	*Hypomontagnella/* *Hypoxylon*	Xiong et al. (2023)
		*Saccharomycetales incertae sedis*		*Starmerella*	Ziuzia et al. (2023); Echeverrigaray et al. (2021);Kędzierska-Matysek et al. (2023); Brudzynski (2021)
	*Schizosaccharomycetes*	*Schizosaccharomycetales*	*Schizosaccharomycetacea*	*Schizosaccharomyces*	Silva et al. (2017); Ziuzia et al. (2023); Xiong et al. (2023); Sinacori et al. (2014)
				*Nematospora*	Silva et al. (2017)
				*Eremothecium*	Silva et al. (2017)
			*Lipomycetaceae*	*Lipomyces*	Silva et al. (2017); Ziuzia et al. (2023); Xiong et al. (2023); Grabowski and Klein (2017); Roxo et al. (2023)
			*Debaryomycetaceae*	*Schwanniomyces*	Silva et al. (2017); Ziuzia et al. (2023); Xiong et al. (2023); Grabowski and Klein (2017); Wei et al. (2022)
				*Debaryomyces*	Silva et al. (2017); Ziuzia et al. (2023); Grabowski and Klein (2017); Echeverrigaray et al. (2021)
			*Metschnikowiaceae*	*Metschnikowia*	Brudzynski (2021); Rodríguez-Andrade et al. (2019); Xiong et al. (2023)
	*Dothideomycetes*	*Pleosporales*	*Torulaceae*	*Torula*	Vázquez-Quiñones et al. (2018); Ziuzia et al. (2023); Xiong et al. (2023); Grabowski and Klein (2017)
			*Didymellaceae*	*Epicoccum*	(Rodríguez-Andrade et al., 2019)
			*Pleosporaceae*	*Alternaria*	Silva et al. (2017); Cinar and Cinar (2021); Kačániová et al. (2012); Brudzynski (2021); Rodríguez-Andrade et al. (2019); Roxo et al. (2023); Kostić et al. (2017)
				*Stemphylium*	Brudzynski (2021)
		*Cladosporiale*	*Cladosporiaceae*	*Cladosporium*	Silva et al. (2017); Kačániová et al. (2012); Brudzynski (2021); Cinar and Cinar (2021); Kiš et al. (2018); Sinacori et al. (2014); Rodríguez-Andrade et al. (2019)
		*Dothideales*	*Saccotheciaceae*	*Aureobasidium*	Silva et al. (2017); Ziuzia et al. (2023); Echeverrigaray et al. (2021); Xiong et al. (2023); Wei et al. (2022); Roxo et al. (2023)
			*Dothioraceae*	*Hormonema*	Balzan et al. (2020)
		*Dothideomycetes incertae sedis*		*Seuratia*	Roxo et al. (2023)
		*Mycosphaerellales*	*Teratosphaeriaceae*	*Coniothecium*	Roxo et al. (2023)
	*Sordariomycetes*	*Hypocreales*	*Hypocreales incertae sedis*	*Cephalosporium*	Roxo et al. (2023)
				*Acremonium*	Kačániová et al. (2012); Rodríguez-Andrade et al. (2019)
			*Clavicipitaceae*	*Metarhizium*	Bovo, Utzeri, Ribani, Cabbri and Fontanesi, 2020a, Bovo, Utzeri, Ribani, Cabbri and Fontanesi, 2020b
			*Nectriaceae*	*Fusarium*	Rodríguez-Andrade et al. (2019); Brudzynski (2021); Kostić et al. (2017); Cinar and Cinar (2021)
		*Xylariales*	*Apiosporaceae*	*Arthrinium*	Sinacori et al. (2014)
			*Sporocadaceae*	*Pestalotiopsis*	Rodríguez-Andrade et al. (2019)
			*Hypoxylaceae*	*Daldinia*	Silva et al. (2017)
		*Sordariales*	*Chaetomiaceae*	*Chaetomium*	Silva et al. (2017)
	*Leotiomycetes*	*Leotiomycetes incertae sedis*	*Myxotrichaceae*	*Skoua*	Brudzynski (2021); Xiong et al. (2023); Rodríguez-Andrade et al. (2019)
				*Oidiodendron*	Brudzynski (2021); Rodríguez-Andrade et al. (2019)
				*Pericystis*	Brudzynski (2021)
				*Bettsia*	Brudzynski (2021); Rodríguez-Andrade et al. (2019); Xiong et al. (2023); Roxo et al. (2023)
		*Helotiales*	*Helotiales incertae sedis*	*Triposporium*	Ziuzia et al. (2023); Xiong et al. (2023); Sinacori et al. (2014); Rodríguez-Andrade et al. (2019)
*Basidiomycota*	*Agaricomycetes*	*Agaricales*	*Cyphellopsidaceae*	*Peyronelina*	Roxo et al. (2023)
	*Tremellomycetes*	*Filobasidiales*	*Filobasidiaceae*	*Naganishia/ Cryptococcus*	Silva et al. (2017); Ziuzia et al. (2023); Xiong et al. (2023); Echeverrigaray et al., 2021, Sinacori et al., 2014, Wei et al., 2022
		*Tremellales*	*Bulleribasidiaceae*	*Dioszegia*	Ziuzia et al. (2023); Xiong et al. (2023); Sinacori et al. (2014); Buzzini et al. (2018); Silva et al. (2017)
				*Vishniacozyma*	Ziuzia et al. (2023); Xiong et al. (2023); Sinacori et al. (2014); Balzan et al. (2020); Silva et al. (2017)
			*Rhynchogastremaceae*	*Rhynchogastrema*	Echeverrigaray et al. (2021)
		*Trichosporonales*	*Trichosporonaceae*	*Trichosporon*	Silva et al. (2017); Ziuzia et al. (2023); Sinacori et al. (2014); Grabowski and Klein (2017)
	*Microbotryomycetes*	*Sporidiobolales*	*Sporidiobolaceae*	*Rhodotorula*	Silva et al. (2017); Ziuzia et al. (2023); Sinacori et al., 2014, Wei et al., 2022
				*Rhodosporidiobolus*	Balzan et al. (2020); Silva et al. (2017);
				*Sporobolomyces*	Ziuzia et al. (2023); Xiong et al. (2023); Balzan et al. (2020)
	*Moniliellomycetes*	*Moniliellales*	*Moniliellaceae*	*Moniliella*	Echeverrigaray et al., 2021, Ziuzia, Janiec, Wróbel-Kwiatkowska, Lazar, & Rakicka-Pustułka, 2023
	*Wallemiomycetes*	*Wallemiales*	*Wallemiaceae*	*Wallemia*	Grabowski and Klein (2017); Balzan et al. (2020); Silva et al. (2017)
	*Exobasidiomycetes*	*Robbauerales*	*Robbaueraceae*	*Robbauera*	Xiong et al. (2023)
	*Malasseziomycetes*	*Malasseziales*	*Malasseziaceae*	*Malassezia*	Balzan et al. (2020); Silva et al. (2017); Grabowski and Klein (2017)
	*Ustilaginomycetes*	*Ustilaginales*	*Ustilaginaceae*		Roxo et al. (2023)
Anamorphic fungi		*Agonomycetales*		*Mycelia*	Kačániová et al. (2012)
*Mucoromycota*	*Mucoromycetes*	*Mucorales*	*Cunninghamellaceae*	*Absidia*	Cinar and Cinar (2021)
*Mucoromycota*	Mucoromycetes	*Mucorales*	*Rhizopodaceae*	*Rhizopus*	Kačániová et al. (2012); Roxo et al. (2023); Felsöciová et al. (2012)
			*Mucoracea*	*Mucor*	Kačániová et al. (2012); Brudzynski (2021); Kostić et al. (2017); Roxo et al. (2023)

**Table 3:** Viruses that can be present in honey.

Realm	Kingdom	Phylum	Class	Order	Family	Genus	Reference
unclassified viruses					unclassified DNA viruses	*Apis mellifera filamentous virus*	Bovo, Utzeri, Ribani, Cabbri and Fontanesi, 2020a, Bovo, Utzeri, Ribani, Cabbri and Fontanesi, 2020b; Papp et al. (2024); Quintana et al. (2021); Gauthier et al. (2015); Revainera et al. (2020); Yañez et al. (2020)
*Monodnaviria*	*Loebvirae*	*Hofneiviricota*	*Faserviricetes*	*Tubulavirales*	*Plectroviridae*	*Suturavirus*	Bovo et al. (2018)
*Varidnaviria*	*Bamfordvirae*	*Nucleocytoviricota*	*Megaviricetes*	*Pimascovirales*	*Ascoviridae*	*Ascoviru TnAV2a/Heliothis virescens ascovirus 3a*	Bovo et al. (2018)
unclassified viruses			*Naldaviricetes*	*Lefavirales*	*Hytrosaviridae*	*Muscavirus/ Musca hytrovirus*	Bovo et al. (2018)
*Duplodnaviria*	*Heunggongvirae*	Uroviricota	*Caudoviricetes*	*Caudovirales*	*Herelleviridae*	*Mooreparkvirus/Mooreparkvirus Lb3381/ Lactobacillus phage Lb338-1*	Bovo et al. (2018)
					*unclassified Caudoviricetes*	*Erwinia phage phiEt88*	Bovo et al. (2018)
*Riboviria*	*Orthornavirae*	*Pisuviricota*	*Pisoniviricetes*	*Picornavirales*	*Dicistroviridae*	*Aparavirus/* *Kashmir bee virus*	Milićević et al. (2018); Yang et al. (2022); Yañez et al. (2020); Ullah et al. (2021)
						*Triatovirus/black queen cell virus*	Milićević et al. (2018); Abou Kubaa et al. (2020); Al Naggar and Paxton (2020); Radzevičiūtė et al. (2017)
					*Iflaviridae*	*Iflavirus/Sacbrood virus*	Yañez et al. (2020); Ullah et al. (2021); McMenamin et al. (2021)
					*unclassified Iflavirus*	*Moku virus*	Remnant et al. (2017)
						*Luteo virus*	Remnant et al. (2017)

The microorganisms found in honey can also be classified according to their usefulness – beneficial microorganisms for bees and humans, and pathogenic microorganisms for bees and humans. The beneficial microorganisms can be usefull in the food industry according to Hamdy et al. (2020); two strains of *Bacillus subtilis* are regarded as cell factories for the production of valuable components for industry (stabilizer, emulsifier, finishing agent, and thickener). Filamentous fungi of the genera *Aspergillus* and *Penicillium* are useful for industrial applications because they generate extracellular materials like acids (citric acid) and enzymes (amylases). These fungi can break down a variety of polymers, including cellulose, hemicellulose, starch, pectin, and also oils and fats (Silva et al., 2017).

Other researchers argued the classification of the microorganisms present in honey based on their ecological niche and place of origin; they divide the microorganisms into bee-foregut, bee-pathogenic microorganisms, and plant-associated microorganisms (Xiong et al., 2023).

An association was made between the microorganisms present in honey and physicochemical parameters such as water activity, titratable acidity, moisture, and also the electrical conductivity of honey (Balzan et al., 2020; Mašková et al., 2020; Xiong et al., 2023). Still, it was found that the microbiological composition of honey could be changed as a result of honey maturation (Wen et al., 2017). After analyzing honey samples collected from stingless bees, Rosli et al. (2020) found that moisture content plays a critical role in fostering bacterial diversity. The bacterial load was higher in samples with greater moisture content than in samples with a lower moisture content. According to Balzan et al. (2020), the composition of fungal and bacterial communities was found to be significantly influenced by moisture alone among the physicochemical data. High environmental moisture levels coupled with incomplete honey ripening can cause higher moisture content, which in turn favors the growth of bacteria and fungi and, implicitly, increases the acidity of honey. Furthermore, microorganism growth can be significantly impacted by pH and a_w_ (Balzan et al., 2020; Wen et al., 2017).

The variety of plants and nectar sources that bees use determines the type of bacteria found in honey (Santorelli et al., 2023). Bees affect the plant nectar with microorganisms that, due to their chemical makeup, prevent goods kept in the hive from going bad (Khan et al., 2021). The process by which nectar is transformed into honey reduces the microbiological composition of the pollen and nectar that bees gather. Honey ripening leads to the removal of a large amount of transient microbial contaminants (Wen et al., 2017).

Acidification, the gradual evaporation of water, and the increase in sugar concentration are the factors that produce a selection for acid-tolerant, osmotolerant, and xerotolerant microorganisms and thus the basic composition of the honey microbiota is obtained (Brudzynski, 2021). Metagenomic analysis reveals that the honey core microbiome shows an overlap between the nectar, pollen, and bee stomach microbiome (Ambika Manirajan et al., 2016). In honey, the most common are *Lactobacillaceae* and *Bacillaceae*, succeeded by *Enterobacteriaceae*, *Bifidobacteriaceae, Acetobacteraceae,* and *Microbacteriaceae* (Bovo et al., 2018). .

### 2.1 Methods for determining the microbiological composition

The microbiological composition of honey can be tested using both culture-dependent and culture-independent methods.

#### 2.1.1 Methods depending on culture

Methods depending on culture involve conventional microbiological methods for the isolation, classification, and enumeration of microorganisms. Such methods were used by many authors including Vázquez-Quiñones et al. (2018) who analyzed 1920 honey samples from 8 states of Mexico, and Kiš et al. (2018) who analyzed 64 honey samples collected from manufacturers in various parts of Croatia.

In order to determine bacteria, specific culture media are used for certain bacterial groups, such as selective culture media (Man-Rogosa-Sharpe agar for lactobacilli, Sabouraud dextrose agar with chloramphenicol or Agar-dichlorane-glycerol – DG 18 for yeasts and molds, Candida agar for *Candida* spp., Mannitol egg Yolk Polymyxin agar (MYP agar) – selective agar for *Bacillus cereus*, Fraser Enrichment Broth for *Listeria*, MacConkey and Violet Red Bile for coliform enumeration etc.) or non-selective media (Nutrient Broth, Columbia Agar Base); certain incubation conditions related to the atmosphere (anaerobic or aerobic) temperature, and other variables can influence bacterial growth. There are also culture media where chemical supplements are used as an additive for the cultivation of nutritionally fastidious microorganisms (Jaradat et al., 2022; Zampieri et al., 2021).

For biochemical and metabolic characterization, several standard tests are performed, such as catalase activity by directly adding a bacterial colony to a drop of 3% H_2_O_2_; if O_2_ gas production occurs then this indicates a catalase-positive strain. One such study was conducted by Adadi and Obeng, 2017a, Adadi and Obeng, 2017b on honey samples from the capital of Ghana. Another standard test is the oxidase test: a test called modified oxidative-fermentative oxidase (Adadi & Obeng, 2017) is used to identify bacteria that produce the electron transport chain enzyme cytochrome *c* oxidase (James F. Collins, 2017). Aerobic oxidase-positive bacteria can use oxygen in respiration as the terminal electron acceptor.

Other investigations to which honey can be subjected to identify the microbiota include fermentation of different substrates (glucose, rhamnose, mannitol, xylose, inositol, sorbitol, arabinose, sucrose, lactose, adonitol, raffinose), nitrate reduction, ornithine decarboxylase, the production of H_2_S, the activity of urease, arginine dihydrolase, tryptophan deaminase, lysine decarboxylase, and proteolytic enzymes, the production of acetoin from glucose, the production of indole from tryptophan, the hydrolysis of β-galactosidase and the use of citrate (as the sole carbon source) (Kešnerová et al., 2016; Wang et al., 2018).

Tests can also be performed to determine the extracellular enzyme activities of the bacteria of interest that were isolated. Such tests were used in the studies, namely: proteolytic test, lipolytic test, cellulolytic test. Adadi and Obeng, 2017a, Adadi and Obeng, 2017b evaluated the bacterial quality of honey from production sites and identified the type of bacteria involved in the contamination of honey collected from the Tamale by performing tests based on culture media, biochemical tests, and Gram stains. The biochemical tests performed included catalase tests, oxidase tests: oxidative-fermentative, modified oxidase; susceptibility to bacitracin and furazolidone, sugar fermentation, oxidase, citrate utilization, indole, motility and urea (Adadi & Obeng, 2017).

#### 2.1.2 Culture-independent methods

Culture-independent methods are based on direct analysis of microbial DNA or RNA from samples, as well as techniques for detecting microbial protein patterns. When compared to the traditional culture method, cutting-edge sequencing tools offer a high level of resolution of honey microbiota composition (Gupta et al., 2019a, Gupta et al., 2019b; Xiong et al., 2023). In the last 30 years different culture-independent methods (CIM) were developed, e.g. PCR-based molecular techniques such as denaturing/temperature gradient gel electrophoresis, single-strand-conformation polymorphism (SSCP), terminal restriction fragment length polymorphisms, quantitative PCR (qPCR) or non-PCR-based molecular techniques such as fluorescence in situ hybridization (FISH) (Su et al., 2012). These methods alongside other developments such as next-generation sequencing (NGS) methods prompted the emergence of many exciting fields such as metatranscriptomics, metagenomics, and metaproteomics. Only a few studies examined the microbial populations of honey using culture-independent methods such as next generation sequencing (NGS) (Bovo et al., 2018; Bovo, Utzeri, Ribani, Cabbri and Fontanesi, 2020a, Bovo, Utzeri, Ribani, Cabbri and Fontanesi, 2020b), or PCR-based molecular techniques such as denaturing/temperature gradient gel electrophoresis (Bednarek et al., 2019) or restriction fragment length polymorphisms (Tsadila et al., 2021). The microbial community was also identified and classified using the amplicon sequencing approach, based on 16S/18S rRNA genes and their associated internal transcribed spacers (ITSs) (Romero et al., 2019; Suphaphimol et al., 2021; Wen et al., 2017)*.*

When compared to the traditional culture method, cutting-edge sequencing tools offer a high level of resolution of honey microbiota composition (Gupta et al., 2019a, Gupta et al., 2019b; Xiong et al., 2023). To identify the isolated bacteria, the sequence of the 16S rRNA gene is amplified from their genetic material (Ngalimat et al., 2019a, Ngalimat et al., 2019b). Amplified fragments are then compared if they show ≥99% resemblance to the nearest identified species with sequences from the GenBank database (Ngalimat et al., 2019a, Ngalimat et al., 2019b). According to Xiong et al. (2023), the metabarcoding methods have a high sequencing depth, are nonselective, and have a high coverage.

For routine identifications, matrix-assisted laser desorption/ionization time-of-flight mass spectroscopy (MALDI-TOF MS) is another culture-independent technique that requires less expertise. The ionization mechanism, as described by Pomastowski et al., 2019a, Pomastowski et al., 2019b, enables the analysis of large biomolecules. This technique is primarily based on the detection of microbial protein patterns (proteomic approach). Milosavljević et al. (2021) used this method to check the microbiological safety and indentify bacteria isolated in sunflower honey from the northeastern area of Serbia*.* Bo et al. (2022) used this method to investigate the microbial contamination in 54 batches of commercial honey from China (Bo et al., 2022). Although the MALDI method for bacteria identification is successful in the clinical field, it is still limited in terms of the identification of species from plants and soil. This constraint might arise from the fact that, in contrast to the BLAST (Basic Local Alignment Search Tool)-type repositories used for the identification of microorganisms with 16S rDNA sequence, the commercial databases used for the MALDI approach contain fewer environmental reference spectra (Pomastowski et al., 2019a, Pomastowski et al., 2019b). Therefore, to improve the identification power of MALDI-TOF MS techniques regarding environmental bacteria, it is necessary to expand the current database of reference spectra, since microorganisms in environmental samples are considered to be more diverse, which implies difficulties in their identification (Pomastowski et al., 2019a, Pomastowski et al., 2019b).

### 2.2 Bacteria present in honey

While the botanical and geographic origin of the plants can influence the microbiological composition of honey, the main species in honey microbiota are species of bacteria from the families *Bacillales* and *Lactobacillales*, followed by *Enterobacteriaceae*, *Bifidobacteriaceae Acetobacteraceae* and *Microbacteriaceae*, and their products, which come from antagonistic interactions of species that can be beneficial for honey activities (Table 1).

#### 2.2.1 Studies about bacterial community composition

Several recent studies from various areas that were conducted on the microbiological composition of honey were considered. In a study recently conducted by Xiong et al. (2023), four types of honey were analyzed: monofloral, wildflower, manuka, and wild, from the microbiological composition point of view. They found that the bacterial community was dominated by *Lactococcus lactis*, common species such as *Serratia*, *Citrobacter*, *Pseudomonas,* and *Cedecea*. Besides these, bacteria such as *Enterobacteriaceae*, *Proteus*, *Asaia*, *Enterobacter*, *Stenotrophomonas, Providencia*, *Bombella*, *Morganella*, *Vagococcus*, *Lactobacillus*, *Delftia*, *Raoultella*, *Bacillus*, *Tyzzerella* were also isolated.

A study conducted by Khan et al. (2020) predominantly identified two bacterial genera *Gluconobacter* and *Lactobacillus*, and occasionally genus *Zymomonas*, and various types of yeast. During the research, they noticed that the bacteria decreased in honey along with the reduction of moisture. The bacteria *Gluconobacter* and *Lactobacillus* disappeared as soon as the required minimum moisture content of roughly 18% was achieved.

Metagenomics studies have an important contribution to completing the information regarding the microbiological community of honey. One such study is the one carried out by Balzan et al. (2020) on 163 honey samples from Italy; the results of this study were obtained using the method based on DNA metabarcoding and indicated that in the microbiota of honey are found mainly phyla *Proteobacteria* with the class *Gammaproteobacteria*, and *Firmicutes* with the class *Bacilli*. The class *Bacilli* was the most representative. The majority of the fungal and bacterial genera/species found in the communities were previously isolated from honey using culture-dependent techniques. Among the bacteria found, there were identified bacteria that produce lactic acid (e.g. *Fructobacillus, Enterococcus, Streptococcus,* and *Lactobacillus*), spore-forming bacteria (e.g. *Clostridium, Paenibacillus,* and *Bacillus*), common food contaminants (e.g. *Acinetobacter*, *Pseudomonas,* and *Propionibacterium*), acetic acid-producing bacteria (*Saccharibacter*) and halophilic bacteria (e.g. *Vibrio*, *Halomonas*, etc.).

Numerous studies analyzed honey mainly in terms of the physiochemical parameters and less from the point of view of microbiological composition. Such studies could explain the presence or absence of different species of microorganisms depending on the physicalchemical results. Such a study that focused on physicochemical analyses and also included microbiological determinatios was the one carried out by Kavanagh et al. (2019) on 131 types of honey from 78 locations in Ireland compared to 8 international samples. For the microbiological analysis, they chose only 9 samples out of the 131 and the data collected indicated that all of the samples used in the microbiological investigation had low levels of aerobic mesophiles. The authors stated that honey can be subject to microbiological contamination also during honey processing and storage due to the manipulation by beekeeper. They analyzed samples stored for 7 days at 27 °C before analysis in comparison with samples kept at 4 °C in the refrigerator until analysis. The results showed that the storage temperature can affect the microbial stability of honey, and more precisely the total number of aerobic mesophilic bacteria. This suggests that storage temperature may affect the microbial stability of honey (Kavanagh et al., 2019). In a study published in 2019 by Pomastowski et al., 2019a, Pomastowski et al., 2019b that was conducted on 20 honey samples from 5 countries (Poland, Italy, Australia, Ukraine, and Portugal), it was found that the physicochemical parameters and the origin of honey are frequently correlated, and they both have a significant impact on the bacterial composition of honey.

#### 2.2.2 Quality and safety indicators - bacteria with influence on health

Regular microbiological tests for hygienic indicator microorganism groups can be used to assess the microbiological quality of honey, such as fungus number, microbe total number, anaerobic spore-forming bacilli, and the number of *Bacillus* spp. (Kędzierska-Matysek et al., 2023). EU legislation (Commission Regulation (EC) No 2073/2005 of 15th of November 2005 on microbiological criteria for foodstuffs) has no specifications on microbiological contamination and on honeybee hygiene. Worldwide, a large number of studies were published on the physicochemical characteristics of honey; however, few studies focus on microbiological contamination, primarily on *Clostridium botulinum*.

From the beginning, it is necessary to specify that for various reasons (e.g. hyperosmolarity, hygroscopicity, antibiotic activities, peroxide content, acidity, etc.), most microorganisms are unable to proliferate or grow in honey. Numerous microorganisms are prevented from growing by the antimicrobial qualities of honey (Maikanov et al., 2019a, Maikanov et al., 2019b). Honey also has a low water activity, which inhibits the growth and survival of bacteria. However, few potential human pathogens were found in honey. Practically, the presence of a significant number of vegetative bacteria in honey could be due to recent contamination.

The existence of different microorganisms can affect the safety and quality of honey. In addition to beneficial microorganisms, the microbiota of honey may contain some species of bacteria, molds, and yeasts that can cause various ailments to the consumer due to the exotoxic products. Contaminant genera and/or pathogens such as *Enterococcus, Escherichia* and *Shigella, Vibrio*, *Propionibacterium, Coprococcus*, *Staphylococcus*, *Faecalibacterium*, *Corynebacterium*, and *Campylobacter* were also identified in the studies carried out from the point of view of microbiological quality. Contamination resulting from human manipulation after honey extraction is highlighted by the existence of the genera *Corynebacterium, Propionibacterium*, *Staphylococcus*, etc. (Balzan et al., 2020). For instance, the majority of *Escherichia coli* strains are safe, but certain serotypes after consumption, can provoke sickness to the host. Certain *Escherichia coli* strains (0157:H7) have the potential to cause renal failure or severe anemia (Adadi & Obeng, 2017).

*Vibrio* can be present when contaminated water comes into contact with bees or when honey is extracted. However, a study by Balzan et al. (2020) also found *Halomonas* in a number of samples, indicating that halophilic bacteria like *Vibrio* and *Halomonas* might be able to survive in honey.

*Shigella* spp. are Gram-negative bacteria that can infect the digestive system and produce a variety of symptoms, including cramps, vomiting, nausea, and diarrhea (Adadi & Obeng, 2017).

*Staphylococcus* species can be transferred from person to product through unhygienic practices and can be a normal component of the microflora of human skin. Infections caused by *Staphylococcus* spp. include pneumonia, sore throat, scarlet fever, endocarditis, meningitis, bronchitis, etc. Contamination of honey with *Staphylococcus aureus* may occur due to inadequate honey handling. The presence of *Salmonella* spp. and coliforms is likely caused by contaminated environments or collection of pollen from inappropriate premises, and also from the use of materials with a deficient decontamination process such as extraction, packaging, and storage. Gram-positive bacteria with the capacity to form spores are found in the genus *Bacillus*. There are sixty species with a very diverse genetic structure. Most of these species are non-pathogenic and some of them are opportunistic pathogenic species (M. S. Silva et al., 2017). These microorganisms are members of the food poisoning-causing and they belong to *Bacillus cereus*, *Bacillus licheniformis*, *Bacillus pumilus*, and *Bacillus majavensis* group. Because the other *Bacillus* species can also produce bacteriocins, they are regarded as safe (M. S. Silva et al., 2017).

The *Clostridium* genus comprises approximately 228 species and subspecies (Tiwari & Nagalli, 2023). From the *Clostridium* species, *Clostridium botulinum* and *Clostridium perfringens* are notable bacteria species that are important for epidemiological research (Grigoryan, 2016). *Clostridium perfringens* is known to produce enterotoxins in the final stages of sporulation. This ubiquitous microorganism is capable of producing approximately 20 different protein toxins (Grenda et al., 2018). The spore-forming, anaerobic*,* Gram-positive *Clostridium perfringens* is commonly isolated from soil and is a normal resident of the microflora of humans and other mammals. This pathogen not only produces small amounts of enterotoxin but also a broad range of hydrolytic enzymes and extracellular toxins. *Clostridium perfringens* strains are distinguished by their ability to produce toxins: enterotoxin (CPE), ι-toxin β-toxin, ε-toxin, NetB, and α-toxin (Maikanov et al., 2019). *Clostridium perfringens* possesses morphological and metabolic characteristics that make it easier to be detected than strains of *Clostridium sporogenes* or *Clostridium botulinum* (Grenda et al., 2018). This pathogen is an opportunistic one that can cause a variety of histotoxic infections in humans, including necrotizing enteritis in animals, gastroenteritis (including non-alimentary and alimentary diarrhea) in adults, and necrotizing enterocolitis in premature infants, which was recently reported. The presence of this microorganism is a sign of environmental contamination with clostridia and fecal spore distribution, as well as the hygienic aspects of food processing distribution (Maikanov et al., 2019a, Maikanov et al., 2019b). In the environment, *Clostridium perfringens* is widespread and may even be a healthy part of the gut microbiota*.* In humans and in a variety of animals, *Clostridium perfringens* can induce enteritis when the balance of the gut microbiome is upset. *Clostridium perfringens* contamination was disclosed by Mauriello et al. (2017) when they analyzed samples from pollen. According to Grenda et al. (2018), there was a 27.5% spore contamination of *Clostridium perfringens* in Polish honey.

So far, honey is considered the only food linked to infant botulism. Because babies under one year of age do not have a fully developed immune system, the presence of *Clostridium botulinum* in honey is considered dangerous. *Clostridium botulinum* is known to be a potent toxin and most of the infant botulism cases are caused by *Clostridium botulinum* strains types A and B, which grow best at temperatures close to human body temperature (Harris et al., 2023). Thus, the detection of the type of *Clostridium botulinum* found in honey is of great interest, as it is essential for ensuring food safety (Guran et al., 2023). The presence of this microorganism in honey could also be due to contamination from bees, nectar, or other external sources (Vázquez-Quiñones et al., 2018). This microorganism enters the hive through contaminated water or even through contact of the product with the ground. There is evidence that the bee gut contains approximately 26-27% Gram-positive bacteria, some of which are *Clostridium* species, including *Clostridium botulinum and Clostridium perfringens* (Vázquez-Quiñones et al., 2018). This organism does not harm bees, but it can cause botulism in humans, particularly in children and people with weakened immune systems (Grigoryan, 2016). The results reported in some studies showed contamination of honey samples from Lithuania and Poland with *Clostridium botulinum* in a percent of 15.35% (Wojtacka et al., 2017) and 21.56% (Wojtacka et al., 2016).

In a study from Kazakhstan carried out on 197 honey samples, the PCR method was used for the identification of anaerobic bacteria and the detection of toxin genes of *Clostridium perfringens* and *Clostridium botulinum* species (Maikanov et al., 2019a, Maikanov et al., 2019b). The results of the analyses indicated the presence of *Clostridium perfringens* in 18 honey samples from the total samples considered in the study, and the presence of *Clostridium botulinum* in one sample (Maikanov et al., 2019a, Maikanov et al., 2019b). mPCR analysis (multiplex PCR) led to the classification of all as type A toxin with the ability to produce toxin α. The analysis of the 16S rDNA gene sequence showed the appearance in 4 samples of other anaerobic species related to *Clostridium botulinum*, namely *Clostridium beijerinckii* and *Clostridium sporogenes* (Maikanov et al., 2019a, Maikanov et al., 2019b).

From the total of 1920 honey samples from Mexico analyzed by Vázquez-Quiñones et al. (2018) to evaluate the microbiological quality, it was found that approximately 60% of the samples met the specifications for the presence of aerobic mesophilic species, while approximately 20% did not meet those for molds and 18.5% did not meet those for yeasts. Regarding coliforms, the samples contained <5 MPN/g. *Clostridium perfringens* was evident in about 10%, with >100 cfu/g. In the case of *Staphylococcus aureus*, no sample was above 100 cfu/g. *Salmonella* spp. was not detected in any of the analyzed samples (Vázquez-Quiñones et al., 2018)*.* Another study published by Fernández et al. (2017) evaluated the microbiological quality of 163 honey samples from Argentina. The study results showed that the analyzed samples did not present any health risk, were free of fecal coliforms, and clostridium spores, as well as free of *Salmonella* and *Shigella* species. Many other studies have reported the absence of *Clostridium* spp. in tested honey samples (Borum & Gunes, 2018; Erkan et al., 2017; Gradvol et al., 2015; Landeka et al., 2022).

In the case of contaminations related to incorrect technological procedures, from honey extraction from hives to honey packaging (including hives, beekeepers, and equipment), the microbial presence could be managed by following the principles of manufacturing and good beekeeping practice (Matović et al., 2018). Contamination with fecal microorganisms can occur through bees that fly into the polluted areas or directly on honey if contaminated instruments are used. Furthermore, a lack of personal hygiene practices and food handler behavior might culminate in cross-contamination (Balzan et al., 2020).

Pollen may be also an important source of contamination with microorganisms (family species of *Enterobacteriaceae, Bacilaceae, Enterococcaceae, Streptococcaceae, Staphylococcaceae, Methylocystaceae, Burkholderiaceae, Acidobacteriaceae, Oxalobacteraceae, Xanthomonadaceae, Micrococcaceae and Acetobacteraceae* etc.*)* (Ambika Manirajan et al., 2016) in bee guts. The bee gut contains 70% Gram-negative bacteria (*Citrobacter, Achromobacter*, *Flavobacterium*, *Klebsiella*, *Escherichia coli*, *Erwinia*, *Proteleus*, *Enterobacter*, and *Pseudomonas*), approximately 27% Gram-positive bacteria (*Bacteridium*, *Bacillus* spp., *Streptococcus* spp., and *Clostridium* spp.), and 1% yeast (Fernández-Estellé et al., 2023). The studies that verified the microbiological quality of honey from various regions of the globe mainly used methods dependent on the culture but also those independent of the culture. In this way the microorganisms were isolated: *Escherichia coli*, *Salmonella* spp., *Staphylococcus aureus*, *Clostridium perfringens*, *Clostridium botulinum*, aerobic mesophilic bacteria, lactic bacteria, coliforms, molds, yeasts.

### 2.3 Molds present in honey

#### 2.3.1 Studies about molds community composition

Honey is a suitable medium for the growth of molds because it contains free amino acids, minerals, and sugars. The presence of molds in honey (Table 2) is common and unavoidable mainly due to improper handling during production and adverse storage conditions (Grabowski & Klein, 2015). Fungi might maintain their vegetative forms, and the growth of fungi leads to the synthesis of mycotoxins. Mycotoxins represent the secondary metabolites of filamentous fungi and they are produced to reduce the incidence of competitors in the environment. They have a toxic effect in low concentrations, both for humans and animals (Silva et al., 2017). Molds have a great ability to survive in nature due to the thermal resistance of spores (Kiš et al., 2018). Mold contamination of honey can happen primarily through nectar, pollen or due to bees, and environmental conditions (air, dust, and soil) (Kačániová et al., 2012). After harvesting honey from hives, secondary cross-contamination can occur through people, equipment, and food handlers. In honey, molds can survive through spores, but they cannot develop (Cinar & Cinar, 2021). In the research carried out on different honey samples, various genera of molds were isolated, such as *Alternaria*, *Aspergillus*, *Acremonium*, *Cladosporium*, *Epicoccum*, *Penicillium*, *Mucor, Fusarium*, *Pestalotiopsis* and *Paecilomyces* (Rodríguez-Andrade et al., 2019).

Cinar and Cinar (2021) identified 11 mold genera, including *Absidia* spp., *Alternaria* spp., *Aureobasidium* spp., *Mucor* spp., *Aspergillus* spp., *Eurotium* spp., *Byssochlamys* spp., *Cladosporium* spp., *Fusarium* spp., *Pacecilomyces* spp., *Penicillium* spp., when they analyzed chestnut honey from Turkey. *Penicillium* spp. was the most frequently identified genus (42%), followed by *Aspergillus* spp. (27%) and *Cladosporium* spp. (13%). Kiš et al. (2018) analyzed 64 honey samples from Croatia and reported that 14.06% of the analyzed samples were contaminated with some mold species and hay honey had the highest number of molds (182 cfu/g).

Kostić et al. (2017) reported that approximately 40% of pollen samples collected from Serbia were contaminated with mold species, including *Penicillium* spp., *Mucor* spp., and *Alternaria* spp. Among the mold genera identified, most species are not harmful to people. Felsöciová et al. (2012), investigated 43 honey samples from Poland and found *Alternaria* spp., *Aspergillus* spp., *Cladosporium* spp., *Fusarium* spp., *Mycelium sterilium*, *Penicillium* spp., *Rhizopus* spp. Due to the fact that honey has great quantities of pollen it was demonstrated that pollen is the main contamination source of honey with *Penicillium* spp. (Sinkevičienė & Amšiejus, 2019).

#### 2.3.2 Quality and safety indicators - molds with influence on health

The existence of some fungal species is problematic for consumption by humans because they can produce highly toxic mycotoxins as part of their metabolism (Kiš et al., 2018). Fungi of the genus *Aspergillus* spp. and *Penicillium* spp. are most commonly found in honey and are the main producers of mycotoxins (M. S. Silva et al., 2017). The conditions required for the growth of fungi are not identical to those required for the production of mycotoxins. The presence of fungi can lead to illness because they produce allergies and infections (Daou et al., 2021).

Fungi of the genus *Aspergillus* can cause bronchopulmonary allergies, asthma, and other forms of aspergillosis. The most pathogenic fungus is *Aspergillus fumigatus*, followed by *Aspergillus flavus*, *Aspergillus terreus*, and *Aspergillus niger*, and inhaled or ingested, spores can cause allergies and asthma. Honey has acidic pH, low moisture, and high concentration of sugars and represents a favorable environment for the growth of *Aspergillus glaucu*s fungi (Machida & Gomi, 2010).

*Penicillium* fungi reproduce vegetatively by spores and are saprophytic fungi capable of growing at water activities of <0.9. They are associated with the production of mycotoxins (aflatoxins, patulins, and ochratoxins). Only the specie *Penicillium marneffei* is pathogenic in humans, causing lung infections, ear infections, and endocarditis in HIV patients (Silva et al., 2017).

Some honey molds, on the other hand, such as *Fusarium, Alternaria*, *Aspergillus*, and *Penicillium* can be allergens (Kostić et al., 2017). Moreover, the presence of certain mold genera in the gastrointestinal tract causes the indirect production of metabolites in the gut microbiota, which can affect immune responses (Cinar & Cinar, 2021). The exceptions in the genera *Alternaria* and *Mucor* are *Alternaria* spp., which is a human allergen, and *Mucor indicus*, which can cause zygomycosis (Kostić et al., 2017). *Penicillium citrinum* was isolated from chestnut honey in the studies reported by Barbosa et al. (2018) and Rodríguez-Andrade et al. (2019). Because *Penicillium citrinum* synthesizes citrinin, which has strong nephrotoxic activity, its presence may indicate a toxicological risk.

The second most common genus found in honey is *Aspergillus. Aspergillus niger*, *Aspergillus candidus*, and *Aspergillus restrictus* are the most frequently reported species. *Aspergillus niger* produces ochratoxin A and fumonisins with toxic effects on the liver, kidney, immune system, and brain. *Aspergillus candidus* which is a potential producer of candidiasis and citrinin, has carcinogenic, genotoxic, and nephrotoxic properties (Rašić et al., 2019).

*Cladosporium* spp. is found in bee guts and is a xerophilic mold that can grow in sweet products and honey, along with *Alternaria* spp. (Kiš et al., 2018). Bovo, Utzeri, Ribani, Cabbri and Fontanesi, 2020a, Bovo, Utzeri, Ribani, Cabbri and Fontanesi, 2020b reported that in Italian polyflora honey, *Aspergillus flavus* was the most representative mold. This is a possible bee pathogen that can cause stonebrood disease. Xiong et al. (2023) identified *Aspergillus flavus* in high quantity in some samples of wildflower, monofloral, and manuka honey.

### 2.4 Yeasts present in honey

Yeasts are a major issue for the honey businesses, particularly osmotolerant yeasts that can cause unwanted fermentation of honey. They can grow in high sucrose concentrations and low pH conditions and the high humidity of the samples favors their development. Honey is fermented by osmotolerant yeasts, which convert glucose and fructose into carbon dioxide and alcohol. The alcohol can be converted into acetic acid in the presence of oxygen which increases the acidity of honey (Ananias et al., 2013; Kiš et al., 2018).

In a recent study in which samples of monofloral, manuka, and wildflower honey were analyzed to identify the microbiological composition, the specific fungal species that were dominant in certain samples were *Bettsia alvei, Yarrowia lipolytica* (monofloral and manuka honey) and *Zygosaccharomyces mellis* (wild honey) (Xiong et al., 2023). Other species of fungi can be found in certain types of wildflower honey, such as *Ascosphaera celerrima, Skoua* spp., *Saccharomyces* spp., and *Zygosaccharomyces rouxii*. 167 fungi were identified in wildflower honey and 80 in monofloral honey, demonstrating that honey has a diverse and distinct fungal genre (Xiong et al., 2023). Beux et al., 2022a, Beux et al., 2022b analyzed Brazilian honey from 3 jataí hives, aiming to isolate and identify the microbiota associated with honeydew and pollen grains. They reported that three yeast species were isolated: *Zygosaccharomyces bailli, Starmerella meliponinorum,* and *Candida magnoliae*.

Balzan et al. (2020) identified by sequencing rRNA16S and ITS2 amplicons in honey gathered from N-E Italy extremophilic yeasts such as *Zygosaccharomyces*, *Dioszegia, Aureobasidium*, *Sporobolomyces*, *Vishriacozyna* and *Candida*, fungi of the genera *Starmerella*, *Candida*, *Malassezia, Wallemia*, *Rhodosporidiobolus*, *Sporobolomyces* and *Hanseniaspora*, or yeast-like fungi *Hormonema* spp. or *Cladosporium* mold. These types of microorganisms were also identified by other authors using culture-dependent methods (Buzzini et al., 2018; Grabowski & Klein, 2017; Silva et al., 2017).

The yeasts found in honey are capable to withstand high concentrations of sugar and acids and although they present an inconvenient problem for the honey sector, they show promise for fermentation processes (*Saccharomyces* spp., *Zygosaccharomyces rouxii*, *Zygosaccharomyces mellis, Schizosaccharomyces* spp., *Rhodotorula* spp., *Hansenula* spp., *Debaryomyces hansenii*, *Oosporidium* spp., *Nematospora* spp., *Lipomyce* spp., *Torulopsis* spp., *Trichosporon* spp., *Aureobasidium pullulans* and *Cryptococcus uzbekistanensis)*. Of these, only *Cryptococcus* species are considered to have a pathogenic effect on humans; the yeast *Cryptococcus neoformans* is a pathogen agent capable of infecting the central nervous system (Silva et al., 2017).

The analysis of honey indicated that yeasts from the genera *Candida*, *Bettsia*, *Eremascus*, *Monascus*, *Metschnikowia*, *Pichia*, *Oidiodendron*, *Saccharomyces*, *Torulopsis*, *Skoua*, *Zygosaccharomyces*, represent the most common group of fungal contaminants. Yeasts from the genera *Zygosaccharomyces, Saccharomyces,* and *Pichia* can grow even at a pH lower than 2, and yeasts from the genera *Ascosphaera*, *Bettsia*, *Eremascus*, *Metschnikowia* survive in conditions where the water activity is very low (up to 0.82) (Rodríguez-Andrade et al., 2019).

### 2.5 Viruses present in honey

Viruses are the most diverse but also the most common group of pathogens. They can cause numerous outbreaks of infectious diseases due to their ability to adapt to new hosts, but also due to their high evolutionary capacity and exponential replication rates. It was demonstrated that insects host a large number of viruses (Wu et al., 2020). Numerous molecular studies were performed in recent years to assess the presence of prevalent viruses in bee colonies around the world (Brown, 2017; de Miranda et al., 2017; Dolezal et al., 2016; Engel et al., 2016; Grozinger & Flenniken, 2019; Li et al., 2017; Locke et al., 2017; McMenamin et al., 2021; Peters et al., 2017; Remnant et al., 2017; Roberts et al., 2018; Schoonvaere et al., 2016; Schoonvaere et al., 2018).

Numerous viruses – the Dicistroviruses (Israeli acute paralysis virus (IAPV), Kashmir bee virus (KBV), Acute bee paralysis virus (ABPV), and Black queen cell virus (BQCV)); the Iflaviruses (Deformed wing virus (DWV), Kakugo virus, Varroa destructor virus-1/DWV-B, Sacbrood virus (SBV), and Slow bee paralysis virus (SBPV)); and taxonomically unclassified viruses (Chronic bee paralysis virus (CBPV) and the Lake Sinai viruses (LSVs)) – that infect bees were isolated thanks to the accessibility of next-generation sequencing approaches (McMenamin & Flenniken, 2018; E. J. Remnant et al., 2017; Hou et al., 2017). They belong to the families *Secoviridae*, *Bunyaviridae*, *Circoviridae*, *Orthomyxoviridae*, *Tymoviridae*, *Flaviviridae*, *Rhabdoviridae*, *Partitiviridae Nodaviridae,* etc. Some of these viruses are associated either with plants (*Secoviridae*, *Tymoviridae*, etc.) or fungi (*Partitiviridae*), and other viruses can be common to both bees, plants (*Secoviridae*), and other insects, etc. These studies shed light on much of the diversity of virus species and strains infecting bees (De Smet et al., 2017; Grozinger & Flenniken, 2019; Simmonds et al., 2017).

However, the literature presents little information regarding the presence of viruses in honey produced by bees (Table 3). Moreover, honey studies are still not carried out to detect viral diseases in bees. Such studies would be useful for beekeepers, especially for the routine control of bee health and to be able to prevent massive losses. In a study by Gauthier et al. (2015) in Switzerland, the *Apis mellifera Filamentous Virus* (AmFV) was isolated from different bee products (honey and pollen) and worker bees that showed the characteristic clinical signs of “milky hemolymph”. This virus has a low impact on the bee being considered a weakly pathogenic virus. This virus was also isolated in a study conducted on honey from 57 beehives in Argentina (Revainera et al., 2020).

Milićević et al., 2018 isolated 2 viruses: the *Black Queen Cell Virus* (BQCV - *Triatovirus nigereginacellulae*) and the *Kashmir Bee Virus* (KBV - *Aparavirus kashmirense*), following a study on 35 samples of honey (30 from Serbia and 5 honey samples from other countries). BQCV was widely present in honey from Serbia, while KBV was found only in imported honey. BQCV was reported in Turkey, Austria, Croatia, France, and China (S. Yang et al., 2022). This type of virus is one of the most widespread bee pathogens responsible for the high mortality of queen pupae (Abou Kubaa et al., 2020; Al Naggar & Paxton, 2020; Radzevičiūtė et al., 2017).

According to Yañez et al. (2020), viruses found in honey can be transmitted from one bee to another via horizontal transmission. KBV and SBV, which were found in honey, pollen, and royal jelly, and AmFV, which was found in pollen and honey, are examples of viruses found in honey that can be transmitted horizontally. Another study about the spread of viruses through honey is the one published by Tantillo et al. (2015) in which they reported that the SBV virus could be transmitted between colonies. Viruses of the genus *Aparavirus* such as KBV and ABPV that cause severe infections in adult bees are transmitted according to Ullah et al. (2021) through honey, pollen, royal jelly, and faeces, as well as Deformed wing virus (DWV - *Iflavirus aladeformis*). DWV can be acquired from other bees horizontally through consumption of contaminated food, mating, bite of a *Varroa destructor* mite, pupal cannibalism, or vertically through infected semen, egg contamination, through infected ovarian tissue (McMenamin et al., 2021).

According to Yañez et al. (2020), some viruses found in honey are part of a complex of related viruses called AKI: Acute bee paralysis virus (ABPV - *Aparavirus apisacutum*), Israeli acute paralysis virus (IAPV - *Aparavirus israelense*) and Kashmir bee virus (KBV - *Aparavirus kashmirense*). This AKI complex was also detected in the genera *Augochlora*, *Bombus*, *Heriades*, *Melipona*, *Andrena* and *Xylocopa*, possibly contaminating genera for bees (Yañez et al., 2020).

Viruses associated with bees can also be present in other insects such as cockroaches, ants (Schläppi et al., 2020), or wasps from which the bee can be contaminated (E. Remnant et al., 2021). Bee colonies are often preyed upon by wasps of the genus Vespula. In addition to stealing honey beehives, wasps also feed on floral sources common with bees (E. Remnant et al., 2021). One of the viruses identified in both insect species is the *Moku* virus, *Luteo* virus (E. Remnant et al., 2021). The infection of the bees in this case can occur by contaminating the honey stored during the raiding of the hive (Loope et al., 2019).

### 2.6 Beneficial microorganisms present in honey

Bees harbour specific microorganisms within every section of their digestive system, and the core of this microbial community consists of various bacterial species. In addition to the gut microbiota, a new lactic bacterial flora consisting of 13 species of *Bifidobacterium* and *Lactobacillus* was discovered (Khan et al., 2020; Raymann & Moran, 2018).

#### 2.6.1 Benefits for the health and protection of the hive

*Bifidobacterium* and *Lactobacillus* (LAB) bacteria are major components of the bee gut microbiota and globally speaking, they are generally conserved in the bee digestive tract (Khan et al., 2020; Raymann & Moran, 2018). It is assumed that due to their fermentation properties, LAB plays a role in the process of transformation of nectar into honey, but also of pollen into bee bread (Nowak et al., 2021). The lactic acid bacterial microbiota plays an essential role in bee health, protecting them from pathogens and contributing to the antimicrobial properties of honey (M. S. Silva et al., 2017).

According to Arredondo et al. (2018), *Lactobacillus kunkeei* protects the hive from possible infections like *Nosema ceranae* and *Paenibacillus larvae*, which is advantageous to the bee colony*.* Other species of lactic acid bacteria *Bacillus megaterium, Bacillus cereus,* and *Bifidobacterium licheniformis*, isolated from honey, have antagonistic action against bee diseases as well as *Paenibacillus* (Abdelbary et al., 2017).

Plants and soil contain bacteria and fungi that can be transmitted by bees to hives through the pollination process. Some of the microorganisms associated with plants that end up in honey have beneficial effects on colonies of bees (Kurek-Górecka et al., 2020). *Actinobacteria* spp. are plant pathogens but many of them protect bees because they produce secondary metabolites that have action against fungal growth. Species from the genera *Enterobacteriaceae* and *Firmicutes* which include *Weissella, Bacillus,* and *Lactobacillus* spp. were initially identified in flowers, and later in both the bee digestive tract and honey (Xiong et al., 2023).

According to a study published by Khan et al. (2020), apart from the advantages they offer to people, the microbes linked to honey are crucial to the survival of bees. They aid in food digestion, colony functioning, immunology, and pollination, and have a negative impact on many infections. Numerous mold species related to bee colonies generate a range of enzymes (leucine aminopeptide, acid phosphatase, β-glucosidase, capillate esteraselipase, and fosamidase) that are involved in protein, lipid, and carbohydrate metabolism. Molds additionally offer antibiotics, organic acids, and other metabolites to hive clusters (K. A. Khan et al., 2020).

#### 2.6.2 Benefits for human health

Previous research showed that beneficial health effects of honey are also due to the presence of endogenous probiotics and prebiotics. Probiotics are live microorganisms that when consumed in appropriate amounts provide beneficial nutritional and physiological health effects (Ebrahimi et al., 2021). The majority of the bacterial species found in honey are xerotolerant, osmotolerant, and acidtolerant and one of the most frequent bacterial species identified in honey samples is *Lactococcus lactis*, a member of the LAB (Brudzynski, 2021). LAB are bacteria that are frequently found in plant materials and they were isolated from bee hives and bee products (Kačániová et al., 2012). Certain byproducts generated by LAB strains have the ability to suppress infections and spoilage organisms while also enhancing the general well-being of hives.

Lashani et al. (2020) analyzed 88 honey samples from different areas of Iran. From these 88 samples, 39 *Lactobacillus* were isolated, of which six came from forest honey, twelve from plain honey, and twenty-one from mountain honey. They were identified by the 16S rDNA gene sequencing method. Among the fructophilic lactic acid bacteria (FLAB), 2 *Fructobacillus fructosus* and 27 *Lactobacillus kunkeei* were isolated. Also, 2 *Lactobacillus paracasei,* 4 *Lactobacillus plantarum*, and 1 isolate each from *Lactobacillus casei, Lactobacillus brevis*, *Lactobacillus fermentum* and *Lactobacillus rhamnosus*. Out of them, one isolation of *Lactobacillus paracasei* and two isolates of *Lactobacillus rhamnosus* completely inhibited every pathogen.

Feizabadi et al. (2021) analyzed honey samples after a one-year storage period and identified viable LAB microorganisms using 16S rRNA sequencing in combination with classical cultivation methods. The results indicate a new species of LAB consisting of eight distinct phylotypes: four *Lactobacillus* and four *Enterococcus* phylotypes. The two most often lactobacilli species reported were *Lactobacillus pentosus* and *Lactobacillus plantarum.*

*Lactobacillus* species*, Clostridium* species*, Bacillus* species, and other lactic acid bacteria (LAB) were isolated from raw honey by Grabowski and Klein (2017). *Lactobacillus plantarum*, *Lactobacillus casei*, *Bacillus subtilis* and *Bacillus velezensis* are antifungal. These bacteria have a strong antifungal effect on yeasts and molds of the genus *Penicillium* and *Aspergillus* spp. (Ramos et al., 2020). Honey samples were used to isolate lactic acid bacteria, which included *Pediococcus pentosaceus, Lactobacillus plantarum*, *Pediococcus acidilactici,* and *Lactobacillus curvatus* that had an inhibitory effect on pathogenic species of *Candida* (Bulgasem et al., 2016) but also on *Saccharomyces*, *Zygosaccharomyces* and *Pichia* spp. (Brudzynski, 2021). Also, LAB can eliminate or inactivate mycotoxins produced by *Aspergillus* (aflatoxin B1 was inactivated and removed by *Lactobacillus rhamnosus*), *Penicillium,* and *Fusarium* (Mathialagan et al., 2018). The participation of LAB bacteria in the inactivation of mycotoxins and their antifungal action protect honey against spoilage and have a role in its preservation and safety (Sadiq et al., 2019).

Ebrahimi et al. (2021) detected *Lactobacillus kunkeei* in honey following PCR sequencing. *Lactobacillus kunkeei* was isolated and tested for antibacterial action, antifungal action, antibiotic resistance, and anti-aflatoxigenic effect. The greatest inhibitory effect of *Lactobacillus kunkeei* was observed on *Escherichia coli*. For *Bacillus cereus,* the inhibition effect was remarkably higher than for *Salmonella enterica* and *Staphylococcus aureus.* Significant in vitro antifungal effects of *Lactobacillus kunkeei* against *Aspergillus flavus* was about 56% and against *Aspergillus niger* about 72%. The LAB isolate showed penicillin susceptibility and semi-resistance to ampicillin, cephalothin, and cefazolin. *Lactobacillus kunkeei* inhibited 19.27 and 20.52% of aflatoxins G1, G2, B1 and B2.

In a study on untreated, unpasteurized natural honey obtained directly from hives in India, Begum et al. (2015) isolated three different bacterial strains (*Pseudomonas* sps. and *Bacillus* sps). Using phylogenetic tree analysis and partial 16S rRNA gene sequence analysis, the identity of the third strain as *Gluconobacter oxydans* was confirmed. *Gluconobacter oxydans* isolated from honey possess probiotic properties.

The lactic acid bacteria *Bacillus cereus, Bacillus megaterium,* and *Bifidobacterium licheniformis*, together with a new strain of *Lactobacillus*, namely *Lactobacillus apis* spp. isolated from honey, possess antagonistic action against human diseases. According to Jia et al. (2020) and Abdelbary et al. (2017), these LAB (*Lactobacillus* and *Bifidobacterium)* performed against important wound pathogens such as vancomycin-resistant *Enterococcus*, *Pseudomonas aeruginosa*, and methicillin-resistant *Staphylococcus aureus*. Moreover, to LAB, up to 90% of the bacteria in the microbiota of honey belong to the species *Bacillus* and *Paenibacillus*, which together produce a broad spectrum of antimicrobial compounds (Brudzynski, 2021).

#### 2.6.3 Benefits for industry

A distinct ecosystem composed of microbes was discovered by other researchers and can be used in the pharmaceutical, cosmetic, and food industries following a study on honey collected from three jataí hives in Brazil (Beux et al., 2022a, Beux et al., 2022b). Four bacteria that are gram-positive cocci (*Staphylococcus vitulinus, Staphylococcus saprophyticus* subsp. *bovis*, and *Staphylococcus pasteuri*) were isolated; two gram-positive spore-forming bacteria (*Bacillus thuringiensis* and *Bacillus pumilus*), and a gram-negative bacteria (*Pantoea agglomerans* – important because can be a possible biological control agent) were detected. Also, a lactic bacteria was identified – *Leuconostoc mesenteroides*, a bacteria that convert glucose molecules into lactate, ethanol, and carbon dioxide which is crucial for preservation and food fermentation. *Bacillus* was the primary bacteria identified in the jatai honey. This study aimed to quantify, separate, and identify the microbiota linked to pollen and honey pots. The authors pointed out that *Bacillus* species could be considered a source of amylase, aiding in the breakdown of sugars and thus assisting in the conversion of nectar into honey. In addition to producing organic acids and antibiotics that work against rival microbes, *Bacillus* can also create digestive enzymes that aid in the pre-digestion of food that was preserved (Beux et al., 2022a, Beux et al., 2022b). The microorganisms found in this study are essential to bee survival.

Hamdy et al. (2020) succeeded in isolating two strains of *Bacillus subtilis* MENO2 and HMNig-2 from honey. According to Hamdy et al. (2020), the two strains have probiotic potential and are regarded as cell factories for the production of levan and other valuable components for industry (stabilizer, emulsifier, finishing agent, and thickener) and pharmaceutical (antihyperlipidemic agent, drug activity extender and plasma substitute) applications (carrier and surface, encapsulating agent for fragrances and flavors).

Hallaj-Nezhadi et al. (2022) isolated *Bacillus velezensis* strain Khuz-3 and *Bacillus safensis* strain Khuz-1 from honey. *Bacillus rugosus* strain Khuz-2 could produce zones of inhibition against the *Candida albicans* human pathogen and against the plant pathogen *Erwinia amylovora, Neurospora crassa, Fusarium oxysparum*, *Rhizobium radiobacter, Botrytis cinerea*. *Bacillus velezensis strain Khuz-3 and Bacillus safensis* strain Khuz-1 had no antimicrobial effect on bacterial affections occurring in humans, including *Klebsiella, Shigella* spp.*, Staphylococcus aureus, Escherichia coli, Escherichia* spp.*, and Bacillus cereus.*

Because *Bacillus* bacteria are able to create antifungal lipopeptides, they have antifungal qualities (Bulgasem et al., 2016). Additionally, these microorganisms have a system in place to naturally eliminate mycotoxins by metabolizing them into innocuous, non-toxic derivatives (Brudzynski, 2021).

In another investigation carried out in Tokyo, 56 samples of fresh honey from several regions of Japan were analyzed (Takatani & Endo, 2021). The honey samples were subjected to analysis one week after harvesting. Lactic acid fructophilic bacteria (FLAB) were found in these samples. These microorganisms can be considered potential probiotic strains with beneficial properties in maintaining good digestive tract health in humans, and they could be contributing to novel strategies toward the rising prevalence of antibiotic-resistant pathogens in human infections, or they could serve as food biopreservatives (Meradji et al., 2023). FLAB species were predominantly found in fresh honey. Most of these were *Fructobacillus fructosus* and *Apilactobacillus kunkeei*. There was no recovery of these bacteria from samples of aged honey, i.e., in samples that were >2 weeks after harvesting (Takatani & Endo, 2021). The study conducted by Zahoor et al. (2021) considered 54 non-*Saccharomyces* and 4 *Saccharomyces cerevisiae* isolates from samples of honey collected from Thailand. Honey sample yeast isolates have great potential as probiotics and as cell factories that can produce useful acids and xylitol. 55 yeast isolates were extracted by Silva et al. (2020) from the honey of stingless bees (Jataí and Iraí). Of the 55 isolates that were found to be satisfactory sources of fermentative yeast species, *Saccharomyces cerevisiae* strains demonstrated the highest potential for producing ethanol.

Apart from contributing to the upkeep of healthier and more productive colonies, these microorganisms may be researched as novel sources of substance for human use. They can be used as biocontrol agents, preservatives, antibiotics, probiotics, and as a supplier of enzymes and antibacterial substances (Beux et al., 2022a, Beux et al., 2022b).

Honey is currently being investigated in the food industry for its ability to inhibit communication and interactions between pathogenic microorganisms. In 2024, Carnicero et al. (2024) investigated the impact of honey's anti-quorum activity on the bacterial cell membrane and they have confirmed that antibacterial effect is due the unique bioactive compunds of honey. Honey provides a number of therapeutic and cosmetic effects for skin, having antimicrobial and wound healing activity, regulating skin pH and also aiding in hair growth (Nikhat & Fazil, 2022).

Abdul Malik et al. (2020) tested the effectiveness of a preparation containing 90% medical grade Manuka honey and 10% glycerine on rosacea and reported significant improvement with this treatment. Similar results have been reported in the case of using honey for seborrheic dermatitis and dandruff.

In 2023, 40 Latvian honey samples were tested for the ability to prevent the formation and activity of gram-negative and gram-positive bacteria biofilms (Skadiņš et al., 2023).The results of the research indicated a promising potential for the use of 11 different types of Latvian honey in the biomaterials industry for wound treatment. Miłek et al. (2023) found that a cream honey enriched with nine vegetable additives had similar potential. The authors have developed functional products that can be recommended as sources of exogenous anti-oxidants. Balázs et al. (2023), Bazaid et al. (2023), Masfufatun et al. (2024), and Shabbir et al. (2023) have also conducted research on antibiofilms.

Pineda et al. (2023) used yellow corn cooking water with yerba mate extract, whey protein concentrate, faxseed flour (a by-product of faxseed oil extraction), and honey to obtain diferent gelled products. The gel with the addition of honey was not sweet, therefore, in addition to obtaining desserts, it can be used to improve physico-chemical properties in fish products (Pourashouri et al., 2020) or in meat products as a fat substitute (Kwon et al., 2021; Pintado et al., 2021). These gels offer an alternative for generating gluten-free products.

Abou Zekry et al. (2020) used honey to obtain nanofibrous dressings used for wound healing. Abou Zekry et al. (2020) tested the wound healing activity of a nanofibrous dressing obtained from pomegranate peel extract, bee venom, polyvinyl alcohol mixed with Manuka honey and freeze-dried powdered honey. The results demonstrated that the dressings obtained in this study had a significant activity against *Staphylococcus aureus* and *Escherichia coli*. It was also observed that by using these manuka honey/ pomegranate/ bee Venom nanofibers dressings, the wound healing rate increased.

Recent studies show that honey is used to obtain hydrogels (pectin-honey hydrogel, chitosan-PVA-gelatin-thyme honey, honey-based alginate hydrogel, chitosan-alginate to incorporate honey and curcumin, etc.) with applications for wound treatment, diabetic ulcer healing and burns. These hydrogels presented the highest antimicrobial activity against the common human infection bacteria including *P. aeruginosa*, *Escherichia coli*, *Staphylococcus aureus, Klebsiella pneumonia* and *Streptococcus pyogenes* (Chotchoungchatchai et al., 2020; Nezhad-Mokhtari et al., 2021)*.*

## 3 The physicochemical parameters that contribute to honey as an antimicrobial agent

Currently, many studies analyzed the composition of honey and investigates the physical and chemical properties that can give rise to its ability to work against various microorganisms responsible for many human diseases (Combarros-Fuertes, Fresno, et al., 2020; Combarros-Fuertes, M Estevinho, et al., 2020; Nolan et al., 2019). There is an increase in interest in honey as a natural resource that can be used in therapies without side effects (Samarghandian et al., 2017). Thus, in recent decades, scientific interest has focused on the antibacterial activity of various types of honey against clinical and foodborne pathogens (Balázs et al., 2023; Combarros-Fuertes, Fresno, et al., 2020; Combarros-Fuertes, M Estevinho, et al., 2020; Faúndez et al., 2023; Skadiņš et al., 2023; Stavropoulou et al., 2022).

The activity that honey has against bacteria is attributed to and influenced by several properties, including its acidity and high viscosity, which is primarily due to a high sugar concentration and low water content (Albaridi, 2019). The presence of hydrogen peroxide and non-peroxidase components, particularly methylglyoxal (MGO), phenolic acids, flavonoids, proteins, peptides, and non-peroxidase glycopeptides, is also thought to contribute to the antibacterial activity of honey (Hossain et al., 2022). To varying degrees, these are all prominent aspects of the antibacterial action of honey, producing either bactericidal or bacteriostatic efficacy (Almasaudi, 2021). These physicochemical parameters (acidity, pH, high sugar concentration, low water content, phenolic acids, flavonoids, proteins, etc.), if considered individually, are not sufficient to demonstrate the antimicrobial activity of honey because they act together. However, the most important parameters that can explain the antibacterial activity of honey are hydrogen peroxide and peptides derived from bees (Cilia et al., 2020).

### 3.1 Acidity of honey

Honey has an acidic pH, which varies between 3.5 and 4.5 due to the organic acids in its composition, which protect it from microbial contamination. A pH of 7.2 to 7.4 is optimal for the development of most microorganisms, so acidic pH is a significant characteristic that contributes to the antibacterial effectiveness of honey (da Silva et al., 2016). The organic acids that can be present in honey are acetic, aspartic, citric, butyric, fumaric, oxalic, galacturonic, formic, gluconic, butyric, glutamic, pyruvic, glutaric, butyric, 2-hydroxybutyric, 2-hydroxy glutaric, isocitric, lactic, methylmalonic, malic, malonic, 2-oxo pentanoic, propionic, quinic, shikimic, succinic, tartaric, and others (da Silva et al., 2016). Organic acids are found in honey in small amounts (0.5%), but they contribute considerably to its antimicrobial and antioxidant activities. The most important and most abundant acid in honey is gluconic acid, which is produced from glucose by the action of glucose oxidase (Soares et al., 2017).

The acidic pH of honey is effective against *Escherichia coli*, *Streptococcus pyogenes*, *Salmonella*, *Pseudomonas aeruginosa*, and yeasts, even though yeasts can survive and multiply at lower pH levels than bacteria. Molds, such as *Aspergillus niger*, can multiply at a pH of 1.2, and the yeast *Candida albicans* grows at a pH of 2 (Yupanqui Mieles et al., 2022). According to Pomastowski et al., 2019a, Pomastowski et al., 2019b electrical conductivity, pH, and acidity levels had a significant impact on the emergence of *Bacillus cereus* strains. The *Bacillus cereus* strains tended to favor honey that was less acidic and more alkaline. Although less pronounced, electrical conductivity, which is correlated with ash content in honey, also had a statistically significant effect. Ash (related to solids and mineral content) and acidity were found to be among the most significant variables associated with honey bacteria levels. Regarding *Bacillus subtilis*, no noteworthy distinctions were noted (Pomastowski et al., 2019a, Pomastowski et al., 2019b).

### 3.2 High carbohydrate content

A percentage between 65 and 80% of the chemical composition of honey is represented by carbohydrates such as glucose, fructose, maltose and sucrose, turanose, melibiose, maltotriose, melezitose, and raphose, etc. (Rajs et al., 2017). This parameter is important for its antimicrobial activity because the high carbohydrate content leads to osmosis, causing bacteria cells to dehydrate and no longer survive in the highly concentrated sugar solution. Honey has a high carbohydrate content and a moisture content of <20%, leading to low water activity (Majtan et al., 2021). Bacterial inhibition varies depending on the type of bacteria and the concentration of honey. The high concentration of sugar in undiluted honey can completely inhibit bacterial growth, but when applied in places where honey can be diluted, the antibacterial action may be lost or limited to specific bacterial species (Hossain et al., 2022).

### 3.3 Water activity (a_w_)

Bacteria need the presence of water in unbound form for development. Molds are the most tolerant to a low value of water activity (0.7), followed by yeasts (0.8) and bacteria (0.9). The low a_w_ in honey does not create optimal conditions for some pathogenic agents, but there are species of fungi with high resistance to a low value of a_w_ (Yupanqui Mieles et al., 2022). The activity of water in honey is low (maximum 0.62), which means that honey does not offer a favorable environment for the development of microorganisms (Albaridi, 2019). The a_w_ value is lower when honey is uncrystallized.

### 3.4 Protein content

Bees secrete four peptides: abaecin, apidaecin, hymenoptaecin, and defensin which have antimicrobial activity. Some studies showed that the defensin peptide (composed of 51 amino acids) acts on molds *Aspergillus* (*flavus* and *niger*), Gram-positive and Gram-negative bacteria and against *Candida albicans* yeasts (Feknous & Boumendjel, 2022). The strong antibacterial activity of defensin-1 in honey is primarily directed against Gram-positive bacteria, including *Staphylococcus aureus, Bacillus subtilis*, and *Paenibacillus* (Yupanqui Mieles et al., 2022). Bílikova et al. (2015) reported that defensin-1 exhibited activity against *Salmonella enterica* and *Pseudomonas aeruginosa* (Gram-negative bacteria). Sojka et al. (2016) studied the action of defensin-1 and reported that it did not show antibacterial activity against planktonic *Escherichia coli*, but significantly reduced the cell viability of *Pseudomonas aeruginosa*. Manuka honey also contains defensin-1, but the antibacterial effect is lessened due to the presence of methylglyoxal (Sojka et al., 2016).

According to some authors, the proteins in honey are derived from pollen or from an enzymatic reaction between bee saliva and plant pollen, and they can also be used as markers for floral honey classification when different types of honey produced by the same bee species are considered (Baroni et al., 2009). Sojka et al. (2016) reported that the highest amount of defensin-1 was found in honey samples of multifloral origin. Antibacterial activity was also demonstrated for the proteins. However, the contribution of defensin-1 to the general antibacterial activity of honey is limited because it is found in rather low concentrations in honey (Bucekova et al., 2019).

### 3.5 Hydrogen peroxide (H_2_O_2_)

Although the enzyme glucose oxidase is present in honey, the high acidity and low water content make it inactive. Upon diluting the honey, glucose oxidase is triggered, converting glucose into H_2_O_2_. According to Brudzynski (2020), honey varieties with darker hues tend to produce higher levels of H_2_O_2_ than those with lighter hues. The maximum level of H_2_O_2_ can vary between 5 and 100 μg H_2_O_2_/g of honey (Hossain et al., 2022).

H_2_O_2_ is thought to be the primary antibacterial component of many types of honey, even though other honey compounds and factors also contribute to its antibacterial activity (Brudzynski, 2020; Brudzynski, 2021; Bucekova et al., 2020). Two types of reactions, enzymatic and non-enzymatic, can break down this compound found in honey. The H_2_O_2_ is converted into two molecules of water and one molecule of oxygen by the primary catabolic enzyme of hydrogen peroxide, catalase. Honey microorganisms and plant nectar are the sources of catalase in honey (Brudzynski, 2020). Hydrogen peroxide can also be transformed into water by other enzymes that contain metals, such as superoxide dismutase and peroxidases. Reactions with vitamin C are part of the non-enzymatic reactions that also lead to the degradation of hydrogen peroxide (Alaerjani et al., 2022).

Farkasovska et al. (2019) analyzed tilia honey samples and reported strong antibacterial activity against *Staphylococcus aureus* and *Pseudomonas aeruginosa*, respectively. An all-encompassing mechanism of the antibacterial action is the production and accumulation of H_2_O_2_. The antibacterial activity of linden honey decreased slightly when H_2_O_2_ was eliminated by adding catalase (Farkasovska et al., 2019).

### 3.6 Methylglyoxal (MGO)

The non-peroxidic antibacterial activity of manuka honey was found to be primarily attributed to MGO (Johnston et al., 2018). Manuka honey has a high concentration of MGO >1000 mg/kg-up to 100 times higher than that of the majority of other honey (Albaridi, 2019; Combarros-Fuertes, Fresno, et al., 2020). Shirlaw et al. (2020) and Lu et al. (2019) have all well-documented the strong antibacterial efficacy of MGO against a variety of bacteria, including *Staphylococcus aureus*, *Escherichia coli*, *Klebsiella pneumoniae*, *Pseudomonas aeruginosa*, *Acinetobacter baumannii,* and *Enterococcus faecalis*.

### 3.7 Phenolic compounds

The antibacterial properties of honey are attributed to the presence of phenolic compounds as well as flavonoids, which are present in honey even in the absence of hydrogen peroxide. Furthermore, the combination of phenolic compounds and hydrogen peroxide has a synergistic effect that contributes to antibacterial qualities (Sawicki et al., 2022). Alvarez-Suarez et al. (2018) reported that there may be a correlation between the high polyphenol content and the antimicrobial activity of *Melipona beecheii* honey.

Kaya and Yıldırım (2021) conducted a study in which the antimicrobial effect of phenolic compounds from honey was examined. According to the published results, floral qualities are positively correlated with the antibacterial qualities of the phenolic compounds it contains. High antimicrobial properties were demonstrated by the study against *Pseudomonas aeruginosa*, *Staphylococcus aureus*, and *Escherichia coli*. Low antimicrobial qualities were shown against *Klebsiella pneumonia* and *Candida albicans*, and moderate antimicrobial properties were shown against *Streptococcus mutans* and *Bacillus subtilis*.

Aumeeruddy et al. (2019) compared 2 honey samples (eucalyptus and commercial honey) and reported that the lower pH of eucalyptus honey compared to commercial honey is a factor responsible for its higher antibacterial activity. In addition, the lower pH of eucalyptus honey is due to the presence of a greater amount of specific organic acids that contribute to its higher antioxidant activity. In fact, a strong positive correlation (*R* = 1000) was observed between pH and the extracellular antimelanogen effect of honey.

Proaño et al. (2021) reported in their study that eucalyptus honey had the ability to act on two human skin infections created by MRSA and *Pseudomonas aeruginosa*. They demonstrated that the antimicrobial activity of eucalyptus honey depended on the presence of H_2_O_2_ and Def-1. Although H_2_O_2_ and Def-1 play an important role in honey's ability to inhibit bacterial growth, the osmotic effect of eucalyptus honey sugars has also been demonstrated.

The polyphenolic compounds present in honey increase the antibacterial activity of honey because they can relate to H_2_O_2_ production, they can reduce Fe (III) into Fe (II) (Fenton reaction) and also, they can produce more potent reactive oxygen species (Romário-Silva et al., 2022). Caffeic acid, galangin, quercetin, and kaempferol, as well as other phenolic compounds present in honey, have shown beneficial effects in the treatment of cardiovascular diseases. They have antioxidant and vasorelaxant properties. Approximately thirty types of phenolic compounds are present in honey, and studies have found that flavonoids exert beneficial effects on the cardiovascular system by inhibiting the activation of blood platelets and by reducing LDL cholesterol levels.

Kassym et al. (2024) tested the action of some polyphenols found in honey, namely 3-phenyllactic acid and p-coumaric acid against the bacterial strain of *Escherichia coli*. The reported results demonstrated that 3-phenyllactic acid was the most effective antimicrobial agent, followed by p-coumaric acid. Thus, the antimicrobial effect of the polyphenols present in honey was demonstrated.

Al-Sayaghi et al. (2022) investigated in their study the antibacterial activities of yemeni sidr honey and manuka honey against *Escherichia coli*. They concluded that methylglyoxal was a bactericidal agent in manuka honey and hydrogen peroxide in yemeni sidr honey, but, other factors contribute to the antibacterial activities of honey, namely: low pH, phenolic and flavonoid content, pressure osmotic, defensin-1, and high carbon‑nitrogen ratio.

Matzen et al., 2018a, Matzen et al., 2018b determined the antibacterial activity of different types of honey and correlated it with the presence of H_2_O_2_ and MGO. In their study they compared the effect of honey on *Staphylococcus aureus*, *Staphylococcus epidermidis*, *Pseudomonas aeruginosa* and *Escherichia coli* with the effect of the pure sugar samples (75% and 15% sucrose) and pure MGO solutions. High concentrations of pure sugar samples alone did not show any inhibition of bacterial growth while pure MGO solutions showed an inhibitory effect on four of the five pathogens tested. They also reported that after treating the honey samples with catalase, an enzyme that inhibits the production of H_2_O_2_, the antibacterial effect of all the Danish honey samples was reduced except for the manuka honey. They also indicated that honey with H_2_O_2_ was more effective than manuka honey in inhibiting dermatophyte fungi and *Candida*.

## 4 Studies on the antimicrobial activity of honey

Microbial agent resistance has grown in recent decades as a result of drugs misuse (Hallaj-Nezhadi et al., 2022; Majkut et al., 2021). An option would be to combine contemporary and traditional medicine, like using honey to combat this type of problem. Honey is a very valuable traditional product with significant antibacterial activity, proven to have bactericidal, fungicidal, virucidal, and antiparasitic properties. Due to the unique combination of vitamins, minerals, organic and other acids, and various antioxidant compounds, honey can be used as a food source as well as for a variety of positive health benefits (Majkut et al., 2021).

### 4.1 Antibacterial activity

Nowadays, antibiotic resistance is one of the most serious public health issues, threatening human health globally (Mohd Kamal et al., 2021). Honey has strong antibacterial properties, making its application in modern medicine an appealing alternative treatment for multidrug-resistant pathogens (Stagos et al., 2018) (Table 4).

**Table 4:** Antibacterial activity of honey.

Antibacterial activity	Type of honey	References
*Acinetobacter calcoaceticus*, *Staphylococcus aureus*, *Pseudomonas aeruginosa* and *Escherichia coli*	heather honey, portobello honey, two blossom honey samples, heather honey (Morayshire), highland honey (Wester Ross) and Portobello Orchard honey (East Lothian)	Deng et al. (2018)
*Staphylococcus aureus, Pseudomonas aeruginosa*	Buckwheat honey (Liaoning, China)	Fyfe et al. (2017)
*Pseudomonas aeruginosa*	Corsican honey	Poli et al. (2018)
*Pseudomonas aeruginosa* and *Staphylococcus aureus*	honey samples from Finland, Sweden, Norway and Denmark	Salonen et al. (2017)
*Staphylococcus aureus, Listeria monocytogenes, Candida albicans, Candida tulpini tropicalis*	cotton honey from Turkey	Combarros-Fuertes et al. (2018)
*Escherichia coli, Pseudomonas aeruginosa, Klebsiella, Proteus, Acinetobacter, Staphylococcus aureus MRSA, Staphylococcus aureus MSSA.*	Egyptian honey – five types of honey (citrus, black seed, mountain, marjoram, and clover honey)	Abd Allah et al. (2018)
*Bacillus subtilis, Pseudomonas aeruginosa, Staphylococcus aureus, Salmonella typhimurium*	honey from Eastern Thailand	Khongkwanmueang et al. (2020)
*Escherichia coli, Pseudomonas aeruginosa, Bacillus subtilis and Staphylococcus aureus*	honey from Malaysia	Syed Yaacob et al. (2018)
*Staphylococcus xylosus, Pseudomonas aeruginosa, Vibrio parahaemolyticus*		Tuksitha et al. (2018)
*Pseudomonas aeruginosa ATCC 10145, Streptococcus pyogenes ATCC 19615*		Al-Kafaween et al. (2020)
*Staphylococcus aureus, Escherichia coli, Klebsiella pneumonia, Pseudomonas aeruginosa, Staphylococcus aureus MRSA*	honey from India	Kirupha et al. (2021)
*Staphylococcus aureus* methicillin-resistant (MRSA*), Staphylococcus aureus* methicillin-susceptible (MSSA)*, Enterococcus fecalis, Escherichia coli, Escherichia coli* producing extended-spectrum β-lactamase (ESBL), *Proteus mirabilis* and *Pseudomonas aeruginosa.*	honey from North West Italy: 26 unpasteurized kinds of honey (5 samples of chestnut honey, 5 samples of dandelion honey, 5 samples of honeydew, 4 samples of lime tree honey, 4 polyfloral mountain honey and 3 rhododendron honey)	Grego et al. (2016)
*Staphylococcus aureus (ATCC 25923 și ATCC 33591), Escherichia coli (ATCC 25922* and *ATCC 35218)*	honeydew honey from Malaysia	Ng et al. (2020)
*Pseudomonas aeruginosa*	honeydew maquis and chestnut grove Corsican honey	Poli et al. (2018)

Antibacterial activity of honey was reported against *Streptococcus pyogenes, vancomycin-resistant enterococci, Salmonella typhi, Escherichia coli, Staphylococcus aureus, Salmonella enterica serovar Typhimurium, Staphylococcus epidermidis, Klebsiella pneumonia, Enterococcus faecalis,* etc. (Mohd Kamal et al., 2021).

Dorota Grabek-Lejko et al. (2018) examined the bactericidal activity of 25 honey samples collected from apiaries in the south-eastern region of Poland. Honey was tested for its antibacterial properties against the laboratory bacteria *Staphylococcus aureus* ATCC 43300 (methicillin-resistant, MRSA) and ATCC 25923 (methicillin-susceptible, MSSA). Their results show that different honey samples may have therapeutic potential against *Staphylococcus aureus*, with buckwheat honey proving to be the most promising.

Acevedo et al. (2017) found that ulmo honey had antibacterial action against the following bacteria strains: *Escherichia coli*, methicillin-resistant *Staphylococcus aureus, Pseudomonas aeruginosa*, *Aeromonas hydrophilia*, *Klebsiella pneumoniae*, and *Salmonella enterica*. In vivo investigations demonstrated that Ulmo (*Eucryphia cordifolia* Cav.) honey may stimulate the monocytic response by regulating antiinflammatory factors, allowing for the regeneration of epithelial cells in burn wounds (Velásquez et al., 2020).

The antimicrobial, nutritional and antioxidant qualities of honey are closely related to its botanical and entomological source, along with the climate and environmental conditions (Stagos et al., 2018; Velásquez et al., 2020)*.* Yang et al. *(*2021*)* *recently conducted a study on many honey samples obtained from four different types of bees from various botanical sources.* The results of the study revealed that the bacterial activity of honey against the pathogens examined (*Staphylococcus aureus, Bacillus subtilis, Escherichia coli*, or *Chromobacterium violaceum*) varied depending on the diversity of bee species and botanical sources of honey *(*Yang et al., 2021*)*.

Manuka honey is another well-known example of honey that has antimicrobial activity against a wide range of pathogens. Manuka honey is derived from the nectar/pollen of manuka plants (*Leptospermum scoparium*), a New Zealand native species. Several investigations were conducted to demonstrate the antibacterial effectiveness of manuka honey against various bacterial infections such as those performed by Hillitt et al. (2017) and Carter et al. (2016). Recent investigations of the antibacterial qualities of various honey varieties produced around the world found that they have comparable or higher antibacterial activity to manuka honey. For example, Stagos et al. (2018) conducted a study on 21 distinct varieties of honey sourced from beekeepers in the Mount Olympus region of Greece. The tested honey types exerted antibacterial activity against *Staphylococcus aureus* and *Pseudomonas aeruginosa* and some of the honey types had a higher antibacterial activity than manuka honey. The antioxidant and antibacterial properties of manuka honey and Polish honey were also compared by Gośliński et al. (2020). The obtained results showed that manuka honey had a significantly high antioxidant capacity when compared to Polish honey. Only honeydew honey was discovered to have antioxidant effects comparable to manuka honey. Honeydew honey has higher antibacterial action against Gram-positive bacteria (Gośliński et al., 2020).

Several scientific investigations examined the antibacterial properties of various varieties of honey, either on their own (Gośliński et al., 2020; Hamid et al., 2018; Hau-Yama et al., 2020; Mduda et al., 2023; Tsavea et al., 2022) or in conjunction with other antimicrobial compounds as a co-adjuvant (Abou Zekry et al., 2020; Combarros-Fuertes, Fresno, et al., 2020; Combarros-Fuertes, M Estevinho, et al., 2020; Gaydhane et al., 2020; Oliveira et al., 2017; Poli et al., 2018; Schuhladen et al., 2021).

Liu et al. (2018) analyzed several medication combinations with various types of honey against *Staphylococcus aureus* biofilms. They have shown that manuka honey plus rifampicin, clindamycin, and oxacillin had a strong synergistic effect in suppressing the proliferation of planktonic cells and preventing biofilm formation by several *Staphylococcus aureus* strains, including methicillin-resistant *Staphylococcus aureus* (MRSA). A study conducted by Oliveira et al. (2017) examined the combined effect of bacteriophages and 13 types of Portuguese honey on *Escherichia coli.* Following the investigation, they concluded that phage and honey treatment together is a potential antibacterial alternative, especially for treating *Escherichia coli* chronic wound infections.

Abou Zekry et al. (2020) examined the use of honey, bee venom, and pomegranate peel extract in conjunction with polyvinyl alcohol to create a new wound dressing. The dressing demonstrated considerable antibacterial efficacy against *Staphylococcus aureus* and *Escherichia coli* in antibacterial testing (Abou Zekry et al., 2020).

Ghalei et al. (2021) created an antibacterial and bioactive scaffold by incorporating honey and a nitric oxide donor, S-nitroso-*N*-acetyl-penicillamine, into polylactic acid nanofibers using a single-jet electrospinning process. Antibacterial experiments found that combining honey and S-nitroso-*N*-acetyl-penicillamine greatly reduced the viability of Gram-positive bacteria *Staphylococcus aureus* and Gram-negative bacteria *Escherichia coli*. The intriguing findings of this study suggest that polylactic acid/honey/S-nitroso-*N*-acetyl-penicillamine nanofibrous scaffolds have considerable promise for application in tissue engineering.

The majority of researches performed until today have proved honey's antimicrobial activity against a variety of microbial strains, including clinical isolates, using in vitro antimicrobial assays. However, few studies have shown honey's antimicrobial activity in vivo, including those involving sublethal doses against pathogens. Therefore, more in vivo research investigating the efficacy of sublethal concentrations of honey against pathogenic biofilms would help increase knowledge of honey's ability to modulate bacterial pathogenicity. Despite the fact that antimicrobial resistance is on the rise around the world, no cases of microbial resistance to honey have been reported, so honey is frequently used as a last resort.

### 4.2 Antifungal activity

The growing occurrence of fungal infections in community and hospital settings, combined with the scarcity of effective antifungal drugs without adverse reactions motivated numerous scientists throughout the world to investigate traditional medicine approaches, and honey began to receive significant attention in recent years. Since 2017, very few studies examined the antifungal activity of honey (Table 5), and the majority of the existing research in this area was concentrated on the antifungal activity of honey against candidiasis (Yupanqui Mieles et al., 2022). Candidiasis is one of the most common infections in the world, caused by the *Candida albicans* and *Candida* non-albicans families (Czernel et al., 2021). *Candida* species are primarily free-living organisms that can be found as commensals in the gastrointestinal and genitourinary systems of humans and also of various species of animals (Ramón-Sierra et al., 2019). Several *Candida* species act as opportunistic infections under particular situations. Although there are medications that are active against multiple *Candida* species, their usage is restricted due to the evolution of resistant strains and the low safety margin for many of these active compounds (Ramón-Sierra et al., 2019). Therefore, candidiasis is an important issue for public health, particularly for patients who are hospitalized. Because new strains of this opportunistic infection that are resistant to traditional antifungal treatments are constantly emerging, it is important to discover novel compounds with efficacy against it, preferably with fewer adverse effects than those already available (Srivastava et al., 2018).

**Table 5:** Antifungal activity of honey.

Antifungal activity	Type of honey	References
*Candida albicans*	stingless bees and honey from the Eastern part of Thailand	Khongkwanmueang et al. (2020)
*Candida albicans, Candida auris, Candida krusei, Candida glabrata, and Candida parapsilosis*	pure medical-grade honey and honey provided from Mook, the Netherlands	de Groot et al. (2021)
*Aspergillus niger, Aspergillus flavus, Aspergillus fumigates, Alterneria alternata, Fusarium solanai, Microsporum canis, Penicillium funiculosium and Rhizopus solanai.*	Pakistani honey (five samples of honey: Ume-e-Shifa, Hamdard, Azka, Marhaba, and raw honey)	Ahmad et al. (2017)
*Aspergillus niger, Aspergillus flavus, Candida albicans, Penicillium chrysogenum, Rhizopus stolonifer, Fusarium oxysporum*	polyfloral honey from different locations in the North-West and the central region of Romania	Vică et al. (2022)
*Candida albicans*	kohgiluyeh and boyer-Ahmad natural honey from Persia	Majidi Poya and Khodavandi (2018)
*Candida albicans, Candida parapsilosis*	honeydew honey from Southern Poland	Czernel et al. (2021)
*Candida albicans, Aspergillus niger*	local Malaysian honey – Tualang honey	Hamid et al. (2018)
*Candida albicans, Candida parapsilosis, Candida glabrata, and Candida tropicalis*	multifloral wild honey and unifloral honey (*Acacia modesta*; *Trachyspermum coptcum*; *Citrus nobili deliciosa*; *Ziziphus* spp.; *Plactranthus* spp.) from Pakistan	Aurongzeb et al. (2019)
*Candida albicans, Aspergillus flavus*	citrus and clover honey from Egypt	Roby et al. (2020)
*Candida albicans*	raw honey from Mexico	Hau-Yama et al. (2020)

Aurongzeb et al. (2019) conducted a study on the efficacy of many types of honey against multidrug-resistant *Candida* species (*Candida albicans, Candida parapsilosis, Candida glabrata*, and *Candida tropicalis*) implicated in skin infections and chronic mucocutaneous candidiasis. They discovered that natural honey samples had antifungal efficacy against all *Candida* species tested at minimum inhibitory concentrations of 3-10% *w*/*v*, with the exception of *Candida glabrata*, which demonstrated resistance to honey samples examined. Anand et al. (2019) investigated the antifungal activity of Agastache monofloral honey, several varieties of manuka honey, Jarrah honey, and tea tree honey against *Candida albicans* (ATCC 10231 and a clinical isolate) and dermatophytes (*Trichophyton mentagrophytes* and *Trichophyton rubrum*). Agastache honey had better antifungal activity than *Leptospermum* honey, implying that Agastache honey products could be created for topical treatment against fungal skin infections (Anand et al., 2019).

Another recent investigation conducted in Romania by Vică et al. (2022) found that honey has antifungal properties against *Candida* spp.*, Aspergillus* spp.*, Fusarium* spp.*, Rhizopus* spp.*,* and *Penicillium* spp.

According to a study on honey from Mexico conducted by Hau-Yama et al. (2020), honey demonstrated antifungal action, inhibiting the growth and multiplication of *Candida albicans* cells and inducing remarkable alterations in the structure and integrity of the cell wall.

It is essential to note that, as previously indicated, the antibacterial activity of honey is impacted by its origin, botanical source, and the species of bee that makes it; similarly, the antifungal activity of honey is influenced by its origin, botanical source, and species of bee that creates it. For example, in the study conducted by Ismail, Abdallah, and Elsharkawy (2021) on five different types of honey from Saudi Arabia, the honey samples did not show antifungal activity but only antibacterial activity; on the other hand, the study conducted by Vică et al. (2022) on several different types of honey from Romania showed both antibacterial and antifungal activity.

Ahmad et al. (2017) found that Pakistani honey (five samples of honey: Ume-e-Shifa, Hamdard, Azka, Marhaba, and raw honey) have significant antifungal tendency against *Penicillium funiculus, Fusarium solanai, Aspergillus flavus, Altrneria alternata, Aspergillus niger, Microsporum canis,* and *Aspergillus fumigate.* Another study conducted by de Groot et al. (2021) on clinical isolates of *Candida albicans, Candida auris, Candida krusei, Candida glabrata, and Candida parapsilosis* from various geographical regions showed that unprocessed honey reduces the growth of pathogenic *Candida* species.

Therefore, depending on its geographical origin and botanical source, honey can be used in the treatment of fungal infections together with antifungal drugs as a less expensive natural remedy, without side effects (Ahmad et al., 2017).

Future clinical trials are required to investigate the impact of honey on infections with other fungi, such as: *Cryptococcus neoformans* (skin lesions)*, Aspergillus* spp.*, Rhizopus* spp.*, Absidia* spp.*, Mucor* spp.*, Malassezia furfur, Trichosporon* spp. (skin and lung lesions)*, Fusarium* spp. (skin lesions), *Pseudallescheria boydii, Scedosporium* spp.*, Alternaria* spp. *(*patients have infected wounds/sores*).* To these are added endemic pathogens that cause a number of mycotic diseases: *Blastomyces dermatitidis, Histoplasma capsulatum, Coccidioidis immitis*, *Paracoccidioidis brasiliensis, Penicillium marneffei.* So far, no medical information has been found about the use of honey in the treatment of these infections caused by fungi, but researchers are confident that honey's antifungal properties will be used in the coming years to treat strains resistant to antifungals (Zammit Young & Blundell, 2023). Future research could focus on the development of wound dressings containing honey or bioactive honey compounds, given that the majority of these pathogens cause skin and lung lesions, as well as the appearance of abscesses with associated symptoms, depending on the organ (De Pauw, 2011)*.* Tissue engineering is a scientific technique that focuses on remolding tissue damage and organ substitutes*.* In tissue engineering, various functional polymer membranes are used to fabricate nanostructured scaffolds for wound dressing usages. Currently there are in vivo and in vitro studies that have shown that the combination of propolis with polymeric wound dressings helps in the wound healing process which can promote their future usage (Alberti et al., 2020). Therefore, more research is needed in this direction.

### 4.3 Antiviral activity

The complex and diversified chemical composition of honey is a significant factor in its extensive therapeutic effect. Its compounds have direct effects on decreasing or removing the risk of severe chronic illnesses in people, including viral disease (Jibril et al., 2019). Although the antimicrobial activity against many fungal and bacterial organisms was widely investigated, the antiviral actions of honey require further investigation so that it may be used in the prevention and treatment of infections caused by viruses (Al-Hatamleh et al., 2020). Several investigations indicate the beneficial properties of honey and its main constituents against a variety of viral infectious illnesses (Mackin et al., 2023) (Table 6). Honey was useful for the prevention and treatment of labial and genital herpes, which are common diseases caused by viruses (Al-Hatamleh et al., 2020). Components of honey can prevent the replication of herpes simplex virus types 1 and 2 (Al-Hatamleh et al., 2020). A study of this type was conducted by Semprini et al. (2017), who investigated the efficiency of kanuka honey treatment against herpes simplex labialis virus. Abdel-Naby Awad and Hamad (2018) conducted another study and found that combining honey with oral acyclovir can provide positive results in children with primary herpetic gingivostomatitis.

**Table 6:** Antiviral activity of honey.

Antiviral activity	References
SARS-CoV-2, Herpes simplex virus type 1, influenza virus A and B, varicella zoster virus	Al-Hatamleh et al. (2020); Asma et al. (2022)
Severe Acute Respiratory Syndrome Coronavirus-2 (SARS-CoV-2) virus or COVID-19	Shaldam et al. (2021);Abedi et al. (2021); Scepankova et al. (2021); Soares et al. (2017); Asma et al. (2022)
human immunodeficiency virus	Asma et al. (2022); Wan Yusuf et al. (2019)
human immunodeficiency virus, SARS-CoV-2	Mackin et al. (2023); Asma et al. (2022)
herpes simplex labialis	Semprini et al. (2017)
herpes simplex gingivostomatitis	Abdel-Naby Awad and Hamad (2018)

Wan Yusuf et al. (2019) investigated the introduction of Malaysian Taulang honey in the dietary regimens of asymptomatic persons with HIV, with particular focus on potential viral load changes, CD4+ T-Helper Lymphocyte count changes and any quality-of-life increases. Researchers found a trend toward lower viral load after administering Tualang honey to asymptomatic HIV subjects, offering validity to the potential role of honey in stimulating the immune system by increasing CD4+ T-helper lymphocyte counts and decreasing viral load in HIV patients (Wan Yusuf et al., 2019). Abedi et al. (2021) conducted research that indicated that honey and its principal components prevent coronavirus entry and replication while also modulating the inflammatory cascade. The strongest interactions with the *SARS-CoV-2* target enzymes include *p*-coumaric acid, ellagic acid, kaempferol, and quercetin, and honey may be considered an effective COVID-19 inhibitor (Shaldam et al., 2021).

The interaction of the primary constituents of honey with structural and/or non-structural proteins in the virus, as well as binding to specific receptors on the virus, appear to have a direct impact on its structural organization (Abedi et al., 2021; Asma et al., 2022). For example, apigenin exhibits antiviral activity against hepatitis B surface antigen and hepatitis B e-antigen. Other bioactive components, like as chrysin, quercetin, kaempferol, and others, may have an antiviral effect by suppressing virus entrance, capture, and replication in human cells (Asma et al., 2022).

Because honey is easily administered to the skin, it can be regarded as a valuable method for treating VZV infection (Asma et al., 2022). According to Abedi et al. (2021) and Asma et al. (2022), honey is capable of fighting against various types of viruses such as herpes zoster virus, rubella virus, respiratory syncytial virus, viral hepatitis virus, rabies virus, rhinoconjunctivitis virus, and others. The mechanisms of antiviral activities of honey and the mechanisms of honey primary components antiviral capabilities are vast and still undetermined. The anti-viral effectiveness of honey and its principal components is frequently coupled with anti-oxidant, anti-inflammatory, anti-resistance, and anti-apoptotic activities by regulating cellular signaling pathways, comparable to other natural compounds like curcumin, resveratrol, calebin A, etc. (Asma et al., 2022). These specific bioactive components of honey, may provide antiviral activity by preventing the entry, entrapment, and replication of viruses. However, the exact mechanism by which honey prevents viral infections is still unknown (Jodidio & Schwartz, 2024). For example, there are currently no honey therapeutic options available to treat infection with one of the most dangerous viruses that has forever changed the course of human history and healthcare: the smallpox virus. In the consulted literature, only one article from 2014 was found (Khan & Dubey, 2014) where it is mentioned that patients infected with smallpox were anointed with honey without providing any other specifications. Other viruses of interest for the development of a possible treatment with honey are: the Ebola virus, the Junin virus that causes Argentine hemorrhagic fever (Kumar et al., 2023). Still, as more multidrug-resistant viruses appear, these mechanisms must be further investigated.

### 4.4 Antiparasitic activity

Despite numerous reports on the antimicrobial properties of various honey types, very few investigations considered its antiparasitic effectiveness (Table 7). Parasites such as *Giardia lamblia, Entamoeba histolytica, Trichomonas vaginalis, Caenorhabditis elegans,* and others are the leading cause of various serious diseases such as dysentery, inflammatory disorders, and sexually transmitted infections, which affect millions of people worldwide each year. Honey from various botanical origins has antiparasitic activity in addition to antiviral, antibacterial, and antifungal effects (Nainu et al., 2021). For example, honey was reported to have nematicidal activity against the embryonic stages of the worm *Caenorhabditis elegans* in a study conducted by Bilal and Azim (2018). The antiparasitic action is attributable to the presence of a variety of beneficial chemicals in honey, especially glycoproteins and glycopeptides, and is based on a variety of anomalies that it causes, particularly in the reproduction process of the parasite, such as egg-laying and hatching issues (Bilal & Azim, 2018). It was also discovered that honey may inhibit the growth of *Entamoeba histolytica* and *Giardia lamblia* trophozoites.

**Table 7:** Antiparasitic activity of honey.

Antiparasitic activity	Type of honey	References
*Plasmodium berghei*	honey produced by stingless bees	Mackin et al. (2023); Laksemi et al. (2023)
*Giardia* and *Trichomonas*	Manuka honey	Sinha et al. (2018)
*Caenorhabditis elegans*	acacia honey from Pakistan	Bilal and Azim (2018)
*Entamoeba histolytica* and *Giardia lamblia*	*Ziziphus spina-christi*, *Acacia nilotica*, *Acacia seyal*, and *Cucurbita maxima* from Saudi Arabia	Mohammed et al. (2019)
*Toxoplasma gondii*	*Capparis spinose* honey from Saudi Arabia	Hegazi et al. (2017)

Mohammed et al. (2019) researched four types of honey (*Acacia nilotica, Ziziphus spina-christi, Cucurbita maxima,* and *Acacia seyal*) for antiprotozoal efficiency against *Entamoeba histolytica* and *Giardia lamblia* in vitro. According to their results, all varieties of honey tested inhibited trophozoite growth. Furthermore, it was discovered that honey consumption increases the number of antibodies as well as levels of cytokines (IFN-, IL-1, and IL-6) in animals infected with *Toxoplasma gondii* (Hegazi et al., 2017).

Laksemi et al. (2023) studied the antiparasitic activities of honey in mice infected with the malaria parasite – *Plasmodium berghei*. They discovered that honey inhibited the malaria parasite and, when combined with the *Citrus aurantifolia* plant extract, had favorable antimalarial action (Mackin et al., 2023). Numerous mechanisms are involved in the action of honey against parasite infections, some of which appear to be aided also by flavonoid compounds, phenolic compounds, and so on (Nainu et al., 2021).

Sinha et al. (2018) study on manuka honey revealed that it can be used as an additional therapy for patients with giardiasis or trichomoniasis. There are now medications on the market that have antiparasitic effects against giardiasis and trichomoniasis, but given the studies that show the emergence of new forms of parasites resistant to drugs, it is fundamental to understand the mechanisms of action of honey with medicinal defining features (El-Senduny et al., 2021; Sinha et al., 2018). According to Hossain et al. (2022) and Cianciosi et al. (2018) the compounds found in honey, such as phenolic compounds, organic acids, enzymes (such as diastase, glucose oxidase, and invertase), minerals (such as potassium, iron, and zinc), along with other minor ingredients, have antiparasitic properties. The aforementioned qualities demonstrate that honey is one of the most versatile bee products, with a diverse set of features and applications that serve as an alternative treatment for a variety of conditions (Sinha et al., 2018). Therefore, honey can be used as a widely available option for parasite control and prevention (Hegazi et al., 2017; Nainu et al., 2021).

## 5 Conclusion

The popularity of honey increased in recent decades due to its possible therapeutic and pharmacological properties. Honey can also contain microorganisms that can come from plants, air, soil, beekeeping, or the equipment they use. These microorganisms have the potential to multiply, modify the product in an undesirable way, and compromise the safety of the honey, therefore it is imperative to control the physicochemical and microbiological parameters of honey. However, honey, which is safe from a microbiological point of view, has antimicrobial properties due to its components (e.g. carbohydrates, hydrogen peroxide, polyphenols, etc.). A detailed analysis of the chemical composition of honey allows estimation of the concentrations of antibacterial components in honey that act synergistically. The studies consulted for this review demonstrate its antiparasitic, anticancer, antiviral, antibacterial, and antifungal properties. Furthermore, the appropriate dose of honey must be determined, as well as how to employ this bee product to treat particular health problems. This information is essential for transferring experimental results from the laboratory bench to the clinic. Future research may focus on creating hydrogels for wounds; dressings with nanofibrillar structure containing honey or bioactive compounds of honey; identifying the mechanism by which honey prevents viral infections; At the same time, depending on the botanical and geographical origin of the honey, it will be possible to investigate the microbiota composition, their antimicrobial action on specific pathogens (*Staphylococcus aureus*, *Salmonella enterica*, *Escherichia coli*, *Pseudomonas aeruginosa* etc.), and a comparison of their antimicrobial action with various antibiotics.

## CRediT authorship contribution statement

**Liliana Luca:** Writing – original draft, Resources. **Daniela Pauliuc:** Writing – review & editing, Writing – original draft, Conceptualization. **Mircea Oroian:** Visualization, Validation, Supervision, Conceptualization.

## Declaration of competing interest

The authors declare the following financial interests/personal relationships which may be considered as potential competing interests:

Daniela Pauliuc reports financial support was provided by Stefan cel Mare University of Suceava. Daniela Pauliuc reports a relationship with Universitatea Stefan cel Mare din Suceava that includes: employment. Daniela Pauliuc has patent pending to No applicable. Not applicable.

## Data Availability

Data will be made available on request.
